# Endogenous auxin maintains embryonic cell identity and promotes somatic embryo development in Arabidopsis

**DOI:** 10.1111/tpj.16024

**Published:** 2022-11-28

**Authors:** Omid Karami, Cheryl Philipsen, Arezoo Rahimi, Annisa Ratna Nurillah, Kim Boutilier, Remko Offringa

**Affiliations:** ^1^ Plant Developmental Genetics, Institute of Biology Leiden Leiden University Sylviusweg 72 2333 BE Leiden The Netherlands; ^2^ Bioscience, Wageningen University and Research Droevendaalsesteeg 1 6708 PB Wageningen The Netherlands; ^3^ Present address: Plus Projects Zwaardstraat 16 2584 TX The Hague The Netherlands; ^4^ Present address: BearingPoint Caribbean Kaya Flamboyan 7 Willemstad Curaçao

**Keywords:** somatic embryogenesis, *Arabidopsis thaliana*, 2,4‐D, *AHL15*, *pWOX2:NLS‐YFP*, IPyA auxin biosynthesis pathway, *YUCCA*, polar auxin transport, *PIN1*, *AUX1*

## Abstract

Somatic embryogenesis (SE), or embryo development from *in vitro* cultured vegetative explants, can be induced in Arabidopsis by the synthetic auxin 2,4‐dichlorophenoxyacetic acid (2,4‐D) or by overexpression of specific transcription factors, such as AT‐HOOK MOTIF NUCLEAR LOCALIZED 15 (AHL15). Here, we explored the role of endogenous auxin [indole‐3‐acetic acid (IAA)] during 2,4‐D and AHL15‐induced SE. Using the *pWOX2:NLS‐YFP* reporter, we identified three distinct developmental stages for 2,4‐D and AHL15‐induced SE in Arabidopsis, with these being (i) acquisition of embryo identity; (ii) formation of pro‐embryos; and (iii) somatic embryo patterning and development. The acquisition of embryo identity coincided with enhanced expression of the indole‐3‐pyruvic acid auxin biosynthesis *YUCCA* genes, resulting in an enhanced *pDR5:GFP*‐reported auxin response in the embryo‐forming tissues. Chemical inhibition of the indole‐3‐pyruvic acid pathway did not affect the acquisition of embryo identity, but significantly reduced or completely inhibited the formation of pro‐embryos. Co‐application of IAA with auxin biosynthesis inhibitors in the AHL15‐induced SE system rescued differentiated somatic embryo formation, confirming that increased IAA levels are important during the last two stages of SE. Our analyses also showed that polar auxin transport, with AUXIN/LIKE‐AUX influx and PIN‐FORMED1 efflux carriers as important drivers, is required for the transition of embryonic cells to proembryos and, later, for correct cell fate specification and differentiation. Taken together, our results indicate that endogenous IAA biosynthesis and its polar transport are not required for the acquisition of embryo identity, but rather to maintain embryonic cell identity and for the formation of multicellular proembryos and their development into histodifferentiated embryos.

## Significance Statement

We show that the induction of embryo identity during 2,4‐D or transcription factor‐induced somatic embryogenesis in Arabidopsis does not rely on an increase in auxin biosynthesis, or the auxin efflux and influx machinery. Rather, auxin biosynthesis and auxin influx are required for the maintenance of embryonic cell identity and formation of pro‐embryos, as well as both auxin biosynthesis and its polar transport for the further development of these pro‐embryos into differentiated somatic embryos.

## INTRODUCTION

The plant hormone auxin, or indole‐3‐acetic acid (IAA), is a major plant growth regulator because it is involved in a wide array of physiological and developmental processes. An important factor determining the developmental role of auxin is its intracellular level, which is affected by *de novo* biosynthesis, transport and metabolism. Together, these three processes generate patterns of auxin maxima and minima in a cell type‐dependent manner (Paque & Weijers, 2016; Vanneste & Friml, 2009).

Recent genetic studies have uncovered several IAA biosynthesis pathways that use tryptophan as main precursor. Of these, the indole‐3‐pyruvic acid (IpyA) pathway has been well‐characterized and is proposed as the main auxin biosynthesis route in *Arabidopsis thaliana* (Arabidopsis) (Tivendale et al., 2014; Zhao, 2018). The IPyA pathway consists of a two‐step reaction. First, tryptophan is converted into IPyA by the TRYPTOPHAN AMINOTRANSFERASE OF ARABIDOPSIS1/TRYPTOPHAN AMINOTRANSFERASE‐RELATED (TAA1/TAR) family of aminotransferases (Stepanova et al., 2008). IPyA is subsequently converted into IAA by the enzymatic activity of the YUCCA (YUC) flavin‐containing monooxygenases (Zhao et al., 2001). Expression of the bacterial tryptophan‐2‐monooxygenase (*iaaM*) auxin biosynthesis gene under the control of a *YUC* promoter in *yuc* knockout mutants (Cheng et al., 2006) or application of IAA (Chen et al., 2014) demonstrated the essential roles of *YUC*‐dependent auxin biosynthesis in Arabidopsis.

Following its synthesis, auxin is transported from source to sink tissues via the phloem and by polar cell‐to‐cell transport (Adamowski & Friml, 2015). The cell‐to‐cell transport of auxin is mainly mediated by plasma membrane‐localized auxin efflux and influx carrier proteins. The PIN‐FORMED (PIN) proteins are auxin efflux carriers, that due to their asymmetric localization at the plasma membrane direct polar auxin transport in plant tissues (Friml, 2010; Habets & Offringa, 2014). IAA enters the cell by passive diffusion or through active import by influx carriers. The generally symmetrically localized AUXIN1/LIKE‐AUX1 (AUX1/LAX) membrane proteins act as permeases that mediate efficient auxin import (Péret et al., 2012; Swarup & Bhosale, 2019).

*De novo* auxin biosynthesis and auxin transport play critical roles in patterning and morphogenesis during zygotic embryogenesis (Lau et al., 2012; Möller et al., 2017). In Arabidopsis, the *YUC1*, *YUC4*, *YUC8*, *YUC*9, *YUC*10 and *YUC11* IPyA pathway genes are expressed in the 8‐ and 16‐cell and globular embryo stages, and *yuc3 yuc9* and *yuc4 yuc9* double mutants, as well as *yuc1 yuc4 yuc10 yuc11* quadruple mutants, exhibit severe embryonic patterning defects (Cheng et al., 2006; Robert et al., 2013). Recently, it has been shown that maternally biosynthesized auxin in the fertilized ovules also provides a source of auxin for the early‐stage zygotic embryo (Robert et al., 2018). However, whether auxin is important directly following fertilization, when the highly specialized, meiotically programmed egg cell is transformed into a totipotent mitotically active embryonic cell (She & Baroux, 2014), is still not known. Similarly, polar auxin transport has been shown to play a vital role in Arabidopsis embryo patterning (i.e. apical–basal and radial embryo axis formation and the establishment of bilateral symmetry by cotyledon initiation) (Friml et al., 2003; Weijers et al., 2005). However, an early role for auxin transport after fertilization has never been established. In addition to the PIN carriers, two auxin influx carriers LAX1 and AUX1 also contribute to formation of the auxin gradients and auxin flow direction during zygotic embryogenesis (Robert et al., 2015; Ugartechea‐Chirino et al., 2010). The stronger embryo defects observed after combining mutations in influx and efflux carriers indicate a cooperative function between auxin influx and efflux carriers in controlling embryo development (Robert et al., 2015).

The ability of a plant cell to acquire totipotency and enter the embryogenesis program is not restricted to the zygote, because embryos can also develop from somatic ovule cells or unreduced gametophytes without fertilization in seeds of apomictic plant species (Hand & Koltunow, 2014; Ozias‐Akins, 2006). In many flowering plants, vegetative somatic cells can also be converted to embryonic cells under appropriate *in vitro* conditions, in a process called somatic embryogenesis (SE). Besides providing a powerful tool for applications in plant biotechnology and plant breeding, including genetic transformation, somatic hybridization, clonal propagation and synthetic seed production, SE offers the potential for understanding cellular and molecular mechanisms that occur during plant embryo initiation and subsequent morphogenesis (Guan et al., 2016; Leljak‐Levanić et al., 2015). Given the importance of SE for plant breeding and propagation, many attempts have been made to understand the molecular basis of this phenomenon in different plant species. Some transcription factor genes have been identified that promote SE. Ectopic expression of a single gene, such as *BABY BOOM* (*BBM*), *LEAFY COTYLEDON 1* (*LEC1*), *LEC2*, *WUSCHEL* (*WUS*) or *AT‐HOOK MOTIF NUCLEAR LOCALIZED 15* (*AHL15*), induces spontaneous SE (Boutilier et al., 2002; Karami et al., 2021; Lotan et al., 1998; Stone et al., 2001; Zuo et al., 2002). SE can also be achieved by exogenous application of plant hormones. Sixty‐five percent of the recent SE protocols use the herbicide 2,4‐dichlorophenoxyacetic acid (2,4‐D), a synthetic analog of the natural auxin IAA, for SE induction (Wójcik et al., 2020). Although 2,4‐D mimics IAA at the molecular level, 2,4‐D is much more stable in plant cells than IAA (Eyer et al., 2016).

Recent research has provided insights into the process of transcription factor‐ and hormone‐induced SE (Horstman et al., 2017; Wójcik et al., 2020), but still the developmental, hormonal and molecular mechanisms governing SE are complex and far from understood. Several studies have shown that 2,4‐D or other exogenously‐applied auxins significantly increase the level of IAA in explants undergoing SE (Awada et al., 2019; Cheng et al., 2016; Ivanova et al., 1994; Jiménez & Bangerth, 2001a; Jiménez & Bangerth, 2001b; Márquez‐López et al., 2018; Michalczuk & Druart, 1999; Vondrakova et al., 2018). IAA accumulation was also found in embryogenic tissues induced by *LEC2* overexpression in Arabidopsis seedlings (Stone et al., 2008). In Arabidopsis, 2,4‐D induces expression of several *YUC* genes in immature zygotic embryo (IZE) explants and embryogenic callus, and higher‐order *yuc* mutants produce fewer somatic embryos per explant compared to wild‐type explants (Bai et al., 2013; Wójcikowska et al., 2013). In addition, ethylene has been reported to have a negative impact on SE in Arabidopsis by reducing *YUC* expression and thereby lowering the auxin levels in embryogenic callus (Bai et al., 2013). Therefore, IAA biosynthesis in embryonic cells appears to play a significant role in SE, although how endogenous IAA promotes SE and whether it is involved in the acquisition of embryo identity is less clear.

Here, we used live‐cell imaging and chemical biology approaches to understand the contribution of endogenous IAA in both 2,4‐D and *AHL15‐*induced SE. Our data show that the induction of embryo identity in somatic explants does not require endogenous auxin biosynthesis, whereas an increase in endogenous auxin levels is essential for maintaining embryo identity and, in conjunction with auxin transport, promoting the development of embryonic cells into histodifferentiated somatic embryos.

## RESULTS

### The *pWOX2:NLS‐YFP* reporter distinguishes different stages of SE in Arabidopsis

IZEs from Arabidopsis are a widely used experimental system for studying 2,4‐D‐induced SE (Gaj, 2001). In our hands, embryonic callus can be efficiently induced on cotyledons of IZEs incubated for 7–9 days of culture on medium supplemented with 4.5 μm 2,4‐D. Following transfer of the explants to 2,4‐D free medium, this embryonic callus develops into globular and eventually cotyledon‐stage somatic embryos (Gaj, 2011; Ikeda‐Iwai, 2002). Overexpression of the *AHL15* gene induces SE on cotyledons of IZEs in the absence of 2,4‐D (Karami et al., 2021). In *p35S:AHL15* cotyledons, the protodermal cells at the adaxial side are converted into embryonic callus at around 6 days after culture. Approximately 2 days later, these embryonic cells develop into globular shaped pro‐embryos.

Here, we used the *pWOX2:NLS‐YFP* reporter (Breuninger et al., 2008) for time‐lapse imaging of embryo initiation in our 2,4‐D‐ and *AHL15*‐induced SE systems. *WOX2* is a member of the *WUSCHEL* (*WUS*) homeodomain gene family and the reporter is expressed in the Arabidopsis zygote, the early embryo proper and the suspensor (Figure S1a). In our 2,4‐D‐based SE system, expression of the *pWOX2:NLS‐YFP* reporter was not detectable in IZE cotyledons within the first 5 days of IZE culture (Figure S1b–d). Relatively weak *pWOX2:NLS‐YFP* activity was first detected in the adaxial regions of cotyledons after 6–7 days. Subequently, 1–2 days later, this signal increased in the areas that formed embryogenic protrusions (Figure S1b–d). No *pWOX2:NLS‐YFP* activity was detected in wild‐type IZE cotyledons cultured in the absence of 2,4‐D (Figure S1b–d). In *p35S:AHL15* IZEs, a relatively weak *pWOX2:NLS‐YFP* signal was first detected 5–6 days after culture in epidermal cells at the adaxial side of *p35S:AHL15* IZE cotyledons, as in the 2,4‐D system (Figure S2a,b,f). Then, 1–2 days later, *pWOX2:NLS‐YFP* expression significantly increased (Figure S2c,f), followed by a reduction in expression in developing globular embryos on days 9–11 (Figure S2d–f). Thus, *pWOX2:NLS‐YFP* is not expressed in cotyledon somatic cells within the first 4 days of culture, but is induced later, becomes highly expressed in dividing embryonic clusters and is then downregulated from the globular stage onward (Figures S1d and S2f). The results indicate that *WOX2* is a good marker for identifying the cell fate transitions to embryo development and marking the developmental stages of SE in both the 2,4‐D and *AHL15*‐induced SE systems.

Based on these observations, we defined three distinct developmental stages during the early process of SE induction from Arabidopsis IZEs: (i) acquisition of embryo identity in somatic cells around day 6; (ii) rapid cell proliferation coinciding with the conversion of embryonic cells to pro‐embryos around day 8; and (iii) the development of pro‐embryos into globular embryos around day 10 of culture in the AHL15‐induced SE system, or following transfer of the explants to hormone free medium in the 2,4‐D‐induced SE system (Figure S1d).

### The IPyA auxin biosynthesis pathway is essential for 2,4‐D‐induced SE

**Figure 1:**
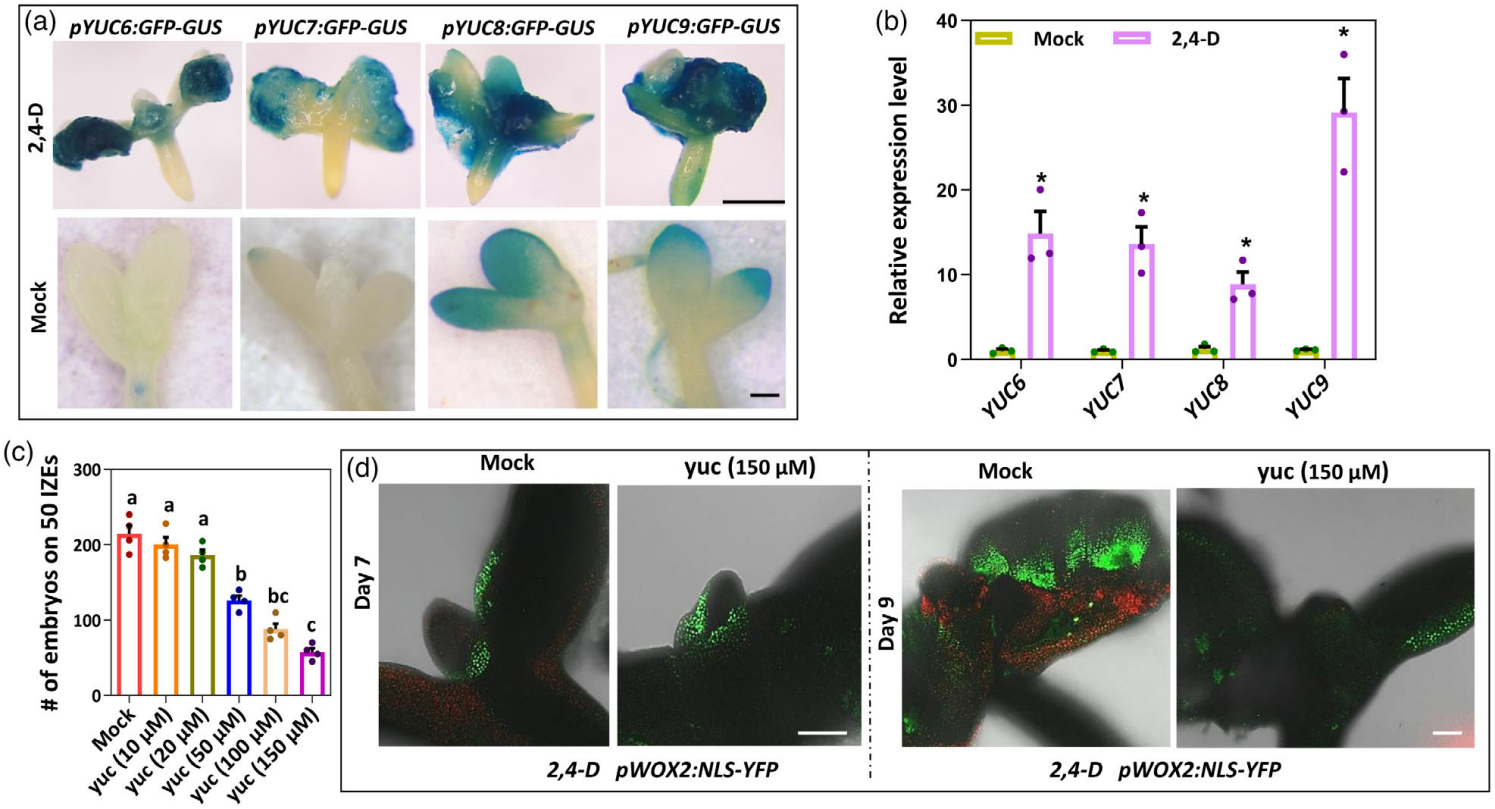
IAA biosynthesis in cotyledon tissues is essential for 2,4‐D induced‐SE. (a) Expression pattern of *pYUC6:GFP‐GUS*, *pYUC7:GFP‐GUS*, *pYUC8:GFP‐GUS* and *pYUC9:GFP‐GUS* reporters in immature zygotic embryos (IZEs) cultured for 8 days on medium with 2,4‐D (upper) or in IZEs cultured for 8 days on medium without 2,4‐D (lower). (b) Relative expression of the *YUC* genes *YUC6*, *YUC7*, *YUC8* and *YUC9* by qPCR analysis in IZEs cultured for 7 days on medium with 5 μm 2,4‐D relative to medium without 2,4‐D. Dots indicate the values of three biological replicates, bars indicate the mean and error bars indicate the SEM. Asterisks indicate significant differences between 2,4‐D and without 2,4‐D (*P < 0.01) as determined by a two‐sided Student's *t* test. (c) Effect of different concentrations of the auxin biosynthesis inhibitor yucasin (yuc) on somatic embryo production in IZEs cultured on medium with 2,4‐D. To allow their survival, IZEs were first precultured for 1 day on medium without yuc and then transferred to medium with yuc. Dots indicate in the number somatic embryos produced on cotyledons of 50 IZEs (*n* = 4 biological replicates), bars indicate the mean and error bars indicate the SEM. Different letters indicate statistically significant differences (*P* < 0.001) as determined by one‐way analysis of variance with Tukey's honest significant difference *post hoc* test. (d) Expression of *pWOX2:NLS‐YFP* in cotyledons of wild‐type IZEs cultured for 7 days (left) and 9 days (right) on 2,4‐D medium without (Mock) and with 150 μm yuc. Images represent an overlay of the green (YFP) and red (chlorophyll) fluorescence. Scale bars = 1 mm.

Previous studies have shown that the *YUC1*, *YUC2*, *YUC4*, *YUC*6, *YUC10* and *YUC11* genes are expressed at the sites of embryo formation during 2,4‐D‐induced SE in Arabidopsis, suggesting that they are responsible for increased IAA biosynthesis in these embryogenic cells (Bai et al., 2013; Wickramasuriya & Dunwell, 2015). However, how this increase in IAA biosynthesis affects the progression of 2,4‐D‐induced SE is not clear. Re‐analysis of the activity of *pYUC*:*GFP‐GUS* reporters in our IZE explants on days 0, 3, 5 and 7 of culture in medium supplemented with 2,4‐D showed that the *pYUC1/2/10:GFP‐GUS* reporters were not or only barely expressed at any time point in culture. However, dynamic expression patterns were observed for the *pYUC4/6/7/8/9/11:GFP‐GUS* reporters, whereas the *pYUC5:GFP‐GUS* reporter showed expression throughout the IZE at all time points (Figure S3). Especially, the *YUC6/7/8/9* reporters were strongly expressed in cotyledon tissue in 7‐day‐old IZE explants cultured on 2,4‐D medium, whereas they barely showed expression in seedlings‐derived from IZE explants that were cultured on medium lacking 2,4‐D (Figure 1a). Quantitative real‐time‐PCR (qPCR) experiments confirmed that expression of the *YUC6/7/8/9* genes was significantly upregulated in IZE explants after 7 days of culture on 2,4‐D medium compared to mock‐treated IZEs (Figure 1b).

Based on these results, we examined the role of the IPyA auxin biosynthesis pathway in 2,4‐D‐induced SE. In view of the strong redundancy between *YUC* genes during zygotic and somatic embryogenesis and the likely lethality of relevant multiple mutant combinations (Bai et al., 2013; Cheng et al., 2007; Robert et al., 2013), we used yucasin (yuc), a specific inhibitor of YUC enzyme activity (Nishimura et al., 2014), to reduce IAA levels during embryogenic callus induction by 2,4‐D. Treatment with 50 μm or higher concentrations of yuc resulted in a significant reduction in the number of somatic embryos (Figure 1c). We propose that the lack of a significant effect of lower yuc concentrations (10 and 20 μm, Figure 1c) is a result of the higher auxin level that is already present in 2,4‐D‐induced explants. Surprisingly, we observed *pWOX2:NLS‐YFP* expression in both untreated and yuc‐treated cotyledons starting from 6–7‐day‐old 2,4‐D‐treated IZE explants (Figure 1d). However, whereas *pWOX2:NLS‐YFP* expression increased in untreated explants 1–2 days later, it decreased in cotyledons of yuc‐treated explants (Figure 1d). These results indicate that endogenous auxin is not required for the initiation of SE, but rather for the maintenance of embryonic cell identity following its acquisition in 2,4‐D‐induced SE.

### Auxin biosynthesis via the IPyA pathway is also essential for AHL15‐induced SE

**Figure 2:**
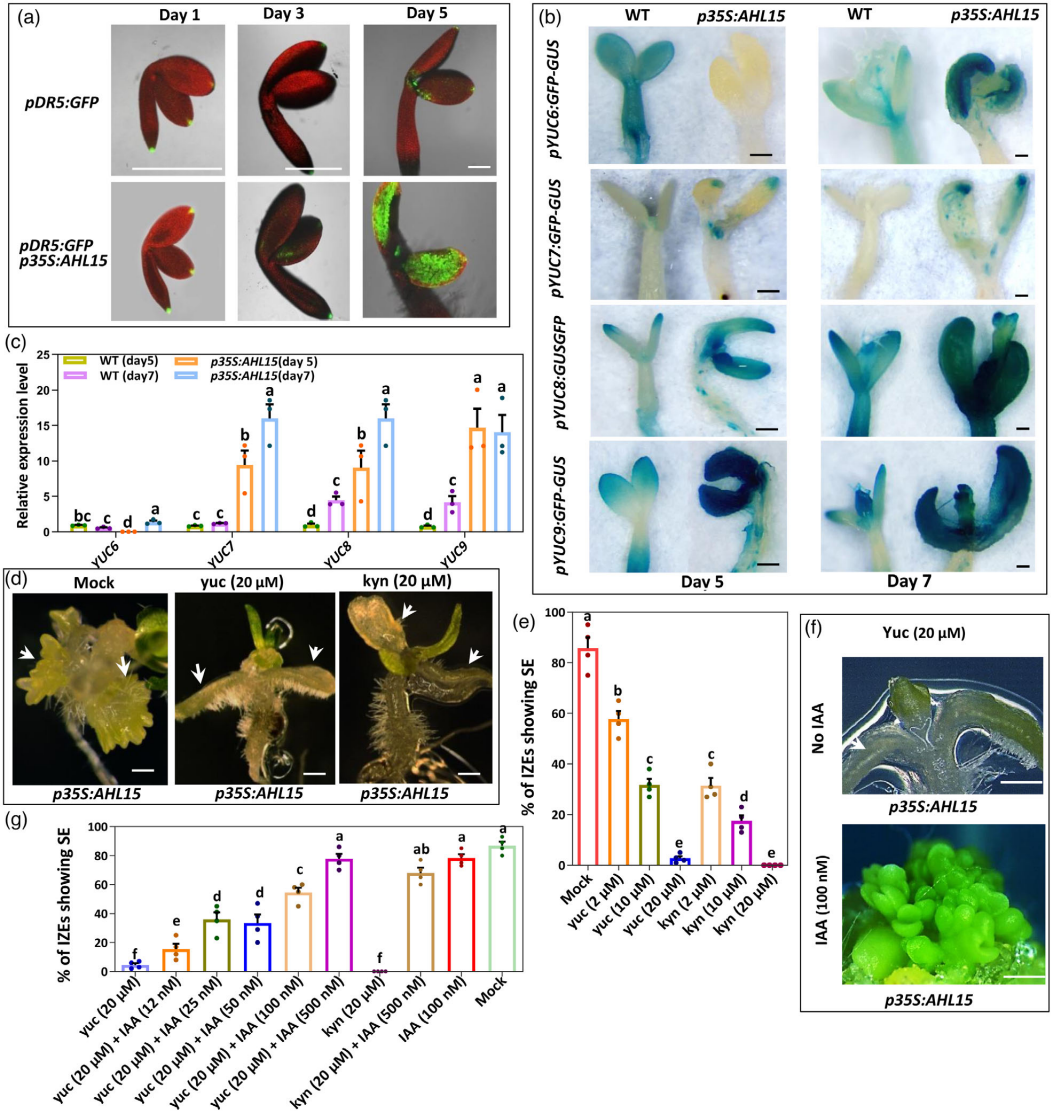
IAA biosynthesis in *35 S:AHL15* cotyledon tissues is essential for AHL15‐induced SE. (a) Expression of the *pDR5:GFP* reporter in wild‐type (upper) or *p35S:AHL15* (lower) IZEs cultured for 1, 3 or 5 days on medium without 2,4‐D. Images represent an overlay of the green (GFP) and red (chlorophyll) fluorescence. (b) Expression pattern of *pYUC6:GFP‐GUS*, *pYUC7:GFP‐GUS*, *pYUC8:GFP‐GUS* or *pYUC9:GFP‐GUS* reporters in wild‐type and *35 S:AHL15* IZEs cultured for 5 days (left) or 7 days (right) on medium without 2,4‐D. (c) Relative expression of the *YUC* genes *YUC6*, *YUC7*, *YUC8* and *YUC9* by qPCR analysis in in wild‐type and *35 S:AHL15* IZEs cultured for 5 or 7 days on medium without 2,4‐D. Dots indicate the values of three biological replicates, bar indicates the mean, and error bars indicate the SEM. Different letters indicate statistically significant differences (*P* < 0.05) as determined by one‐way analysis of variance with Tukey's honest significant difference *post hoc* test. (d) The phenotypes of *p35S:AHL15* IZEs cultured for 2 weeks on medium without 2,4‐D and with or without (Mock) 20 μm of the auxin biosynthesis inhibitor yuc (middle) or kyn (left). White arrowheads indicate the adaxial side of cotyledons. (e) Effect of different concentrations of yuc and kyn on the efficiency of somatic embryo induction on cotyledons of *p35S:AHL15* IZEs. (f) The phenotypes of *p35S:AHL15* IZEs cultured for 2 weeks on medium without 2,4‐D, but with 20 μm yuc and with or without (No IAA) 100 nm IAA. White arrowheads indicate adaxial side of cotyledons. (g) Exogenous IAA treatment restores yuc‐ or kyn‐impaired SE on cotyledons of *p35S:AHL15* IZEs. Dots in (e) and (g) indicate the percentage of *p35S:AHL15* IZEs producing somatic embryos (*n* = 4 biological replicates, with 50 IZEs per replicate), bars indicate the mean, error bars indicate the SEM and different letters indicate statistically significant differences (*P* < 0.001) as determined by one‐way analysis of variance with Tukey's honest significant difference *post hoc* test. (a, b, d, f) Scale bars = 1 mm.

Next, we studied the role of auxin biosynthesis in AHL15‐induced SE. AHL15‐induced SE occurs in the absence of exogenous 2,4‐D, which allowed us to follow the spatiotemporal endogenous auxin dynamics in *p35S:AHL15* cotyledon tissues using the auxin responsive *pDR5:GFP* reporter (Benkova et al., 2003). Time‐lapse analysis showed that *pDR5:GFP* activity did not differ between wild‐type and *p35S:AHL15* cotyledons during the first 3 days of culture (Figure 2a). However, 1–2 days later, *pDR5:GFP* reporter expression markedly increased throughout the entire *p35S:AHL15* cotyledon, whereas no or a much lower GFP signal was observed in wild‐type explants (Figure 2a). These results suggested that AHL15 induces auxin responsive gene expression in cotyledons of IZEs.

Comparison of the expression of *pYUC:GFP‐GUS* and *pYUC:NLS‐3xGFP* reporters in *p35S:AHL15* or wild‐type IZE cultures at 5 or 7 days of culture on hormone free medium did not reveal obvious differences in activity of the *YUC2/4/5/11* (Figure S4a,b) promoters. *YUC1/3/10* promoters were not or only barely expressed at 5 or 7 days of culture (Figure S4a,b), whereas *YUC7/8/9* promoter activity increased in *p35S:AHL15* explant cotyledons compared to wild‐type explant cotyledons (Figure 2b). Interestingly, in 5‐day‐old IZE explants, *pYUC6:GFP‐GUS* expression was not detected in *35 S:AHL15* cotyledons, whereas it was expressed in wild‐type explants (Figure 2b). However, after 7 days of culture, expression of this reporter was strongly upregulated in *p35S:AHL15* cotyledons (Figure 2b), whereas it was reduced in wild‐type cotyledons. qPCR experiments showed that expression *YUC6/7/8/9* genes was significantly upregulated in *p35S:AHL15* (Figure 2c) compared to wild‐type IZE explants after 5 and 7 days of culture. Based on these results, we speculate that the increase in *pDR5* expression in *p35S:AHL15* cotyledons in the same time frame might be caused by the induction of *YUC6/7/8/9* gene expression.

We did not detect a significantly higher level of *pDR5:GFP* activity or *pYUC6/7/8/9* reporter expression in hypocotyl or root tissues of *p35S:AHL15* explants compared to wild‐type explants. This implies that *YUC6/7/8/9* genes are specifically upregulated in cotyledon tissues, causing a cotyledon‐specific increase in auxin response in *p35S:AHL15* explants.

Treatment with the yuc auxin biosynthesis inhibitor completely inhibited somatic embryo induction in *p35S:AHL15* IZE explants. The same results were obtained with l‐kynureine, an inhibitor of the TAA1/TAR aminotransferases that mediate the first step in the IPAy auxin biosynthesis pathway, upstream of the YUC proteins. Compared to 2,4‐D‐induced SE, inhibition of somatic embryo formation occurred at relatively low (20 μm) yuc or kyn concentrations (Figure 2d,e). The negative effect of yuc and kyn on AHL15‐induced SE could be rescued by exogenous application of 12–500 nm IAA, at concentrations that did not inhibit SE in the absence of yuc or kyn (Figure 2f,g). These results, together with the observed induction of *YUC6/7/8/9* gene expression, suggest that the IPyA pathway‐mediated IAA biosynthesis is required for AHL15‐induced SE. The rescue of yuc‐ or kyn‐inhibited SE by exogenous application of relatively low auxin concentrations (Figure 2f,g) suggests that AHL15‐induced SE is hypersensitive to changes in auxin levels, implying that a relatively small increase in auxin levels is sufficient for AHL15‐induced SE.

### YUC‐dependent auxin biosynthesis during SE maintains embryonic identity

**Figure 3:**
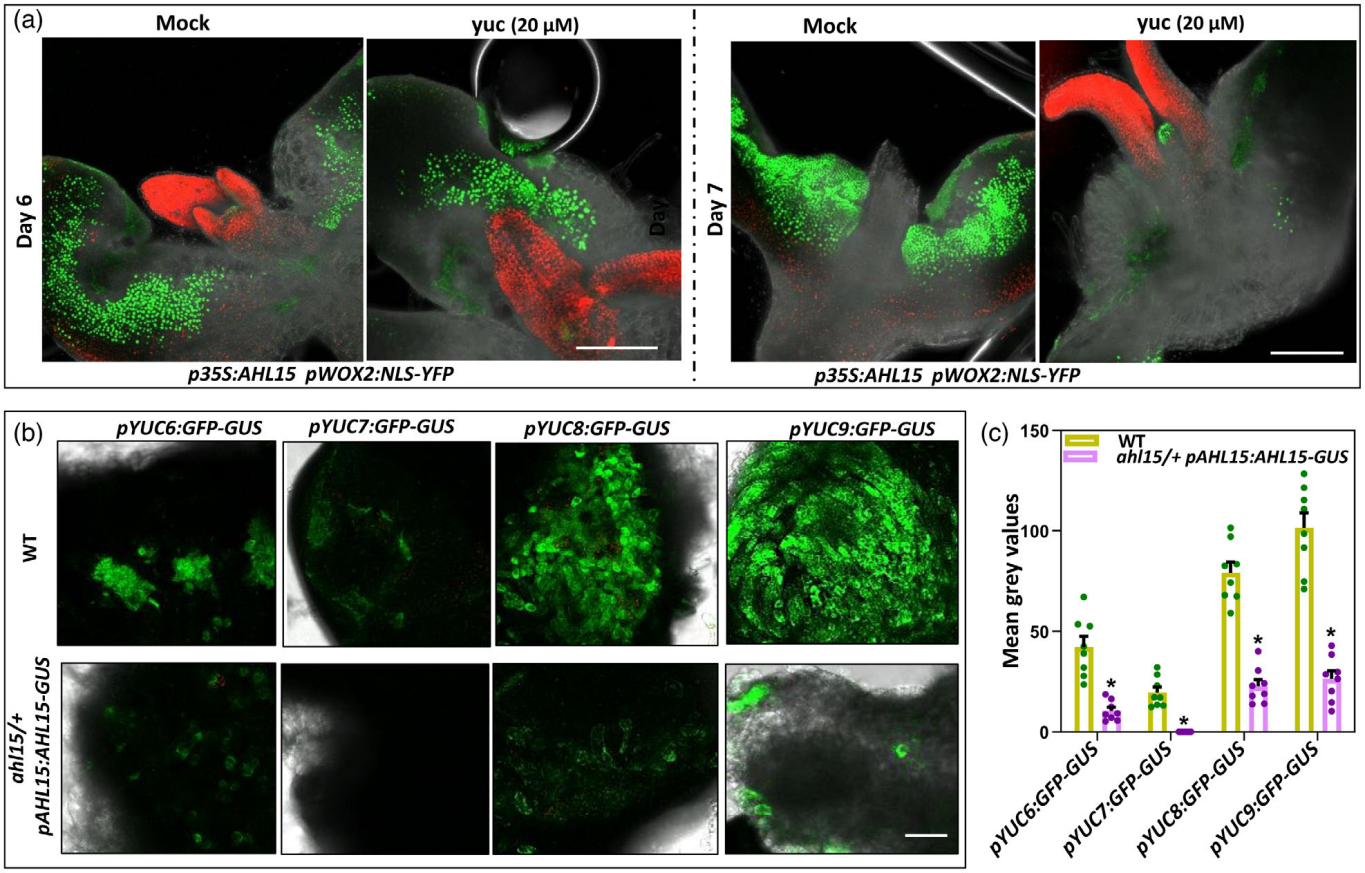
YUC‐mediated auxin biosynthesis is required for the maintenance of embryonic cell identity. (a) The expression of *pWOX2:NLS‐YFP* in cotyledons of germinating *p35S:AHL15* IZEs after 6 days (left) and 8 days (right) of culture on medium with or without (Mock) 20 μm yuc. Images represent an overlay of the green (YFP) and red (chlorophyll) fluorescence. (b) Expression of the *pYUC6:GFP‐GUS*, *pYUC7:GFP‐GUS*, *pYUC8:GFP‐GUS* or *pYUC9:GFP‐GUS* reporters in cotyledons of wild‐type (WT) or *ahl15/+ pAHL15:AHL15‐GUS* IZEs cultured for 8 days on 2,4‐D medium. Scale bars = 0.5 mm. (c) Quantification of GFP intensity (mean gray values) in *pYUC6:GFP‐GUS*, *pYUC7:GFP‐GUS*, *pYUC8:GFP‐GUS* or *pYUC9:GFP‐GUS* in cotyledons of wild‐type (WT) or *ahl15/+ pAHL15:AHL15‐GUS* IZEs cultured for 8 days on 2,4‐D medium. Dots indicate the values of eight biological replicates, bars indicate the mean and error bars indicate the SEM. Asterisks indicate significant differences from WT (*P < 0.01) as determined by a two‐sided Student's *t*‐test.

The similar timing of induction of *pDR5:GFP* (Figure 2a) and *pWOX2:NLS‐GFP* (Figure 3a) reporter expression in *p35S:AHL15* cotyledons suggested that the enhanced auxin biosynthesis and response coincide with the acquisition of embryo identity in cotyledon tissues. Expression of the *pWOX2:NLS‐YFP* reporter was detected in the cotyledons of both 5–6‐day‐old untreated and yuc‐treated *p35S::AHL15* IZE explants (Figure 3a). However, 1–2 days later, when *pWOX2:NLS‐YFP* expression increased in the untreated control, *pWOX2:NLS‐YFP* expression decreased or disappeared in cotyledons of yuc‐treated *p35S::AHL15* IZEs (Figure 3a). These results indicate that the cotyledon cells in yuc‐treated *p35S:AHL15* IZE explants initially acquire embryo identity, although these newly induced‐embryonic cells are not stable and quickly return to the non‐embryogenic state in the absence of auxin biosynthesis. This was also observed for 2,4‐D‐induced SE and implies that endogenous auxin production mediated by the IPyA pathway is not required for the acquisition of embryo identity, but mainly contributes to the maintenance of embryonic identity following its acquisition and somatic embryo development.

### *AHL* genes are required for induction of *YUC* genes during 2,4‐D‐induced SE

We previously reported that *AHL15* is highly expressed in 2,4‐D‐induced embryogenic tissues and that expression of a *pAHL15:AHL15‐GUS* fusion in the *ahl15*/+ heterozygous or *ahl15* homozygous mutant background respectively inhibits 2,4‐D‐induced SE or arrests zygotic embryogenesis. Expression of the AHL15‐GUS fusion in these mutant backgrounds leads to a dominant‐negative effect that overcomes the functional redundancy between *AHL15* and other *AHL* family members, and thus leads to *ahl* loss‐of‐function (Karami et al., 2021). The expression of *YUC6/7/8/9* was significantly reduced in cotyledons of 2,4‐D‐treated *ahl15/+ pAHL15:AHL15‐GUS* IZEs compared to wild‐type IZEs (Figure 3b,c), suggesting that these *YUC* genes may act downstream of AHL15 during 2,4‐D‐induced SE. The significantly lower expression of the *pDR5:GFP* reporter in *ahl15 pAHL15:AHL15‐GUS* zygotic embryos (Figure S5b) compared to wild‐type *pAHL15:AHL15‐GUS* embryos (Figure S5a) suggests that *AHL* genes also play a role in stimulating IAA biosynthesis in zygotic embryos, possibly through induction of *YUC* gene expression.

### Auxin efflux is required for proper development and patterning of somatic embryos

Auxin efflux carriers play an important role in zygotic embryo patterning (Friml et al., 2003), but their role in the initiation of zygotic embryogenesis is still unclear. In our hands, 2,4‐D‐induced SE arrests with the formation of pro‐embryos after approximately 10 days of culture on 2,4‐D containing medium (Figure S1). Patterning of these pro‐embryos into somatic embryos only occurs after explant transfer to 2,4‐D free medium, suggesting that the transition from a pro‐embryo to a histodifferentiated (patterned) embryo is inhibited by 2,4‐D.

**Figure 4:**
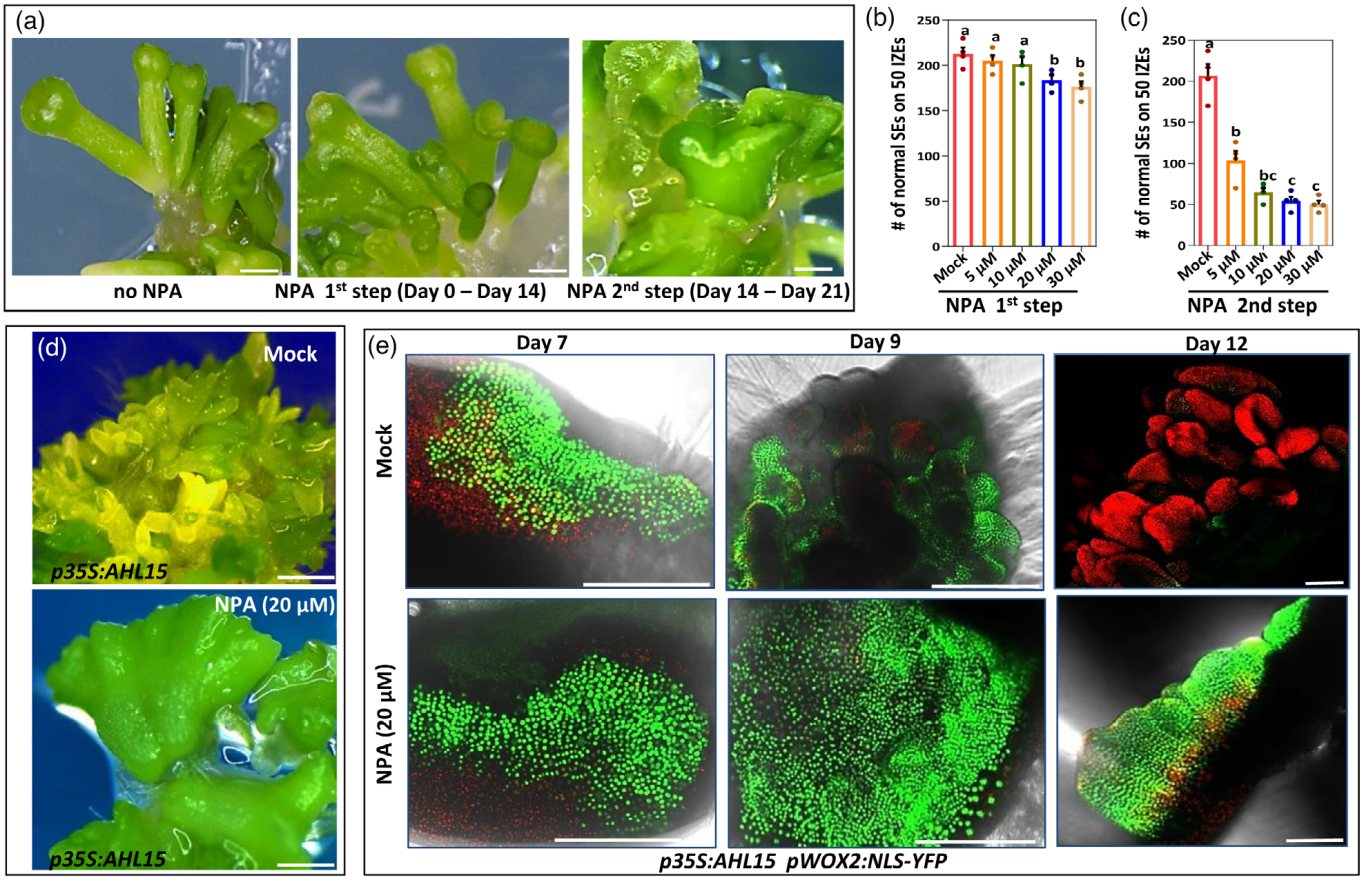
Auxin efflux is required for the proper development of embryonic cells into somatic embryos. (a) The phenotype of somatic embryos formed on cotyledons of wild‐type IZEs that were first grown for 2 weeks on 2,4‐D medium (left) or on 2,4‐D medium with 20 μm NPA (middle) and subsequently cultured for 1 week on medium without 2,4‐D or for 2 weeks on 2,4‐D medium and subsequently for 1 week on medium without 2,4‐D and with 20 μm NPA (right). (b) The number of non‐fused (normal) somatic embryos (SEs) per 50 IZEs that were first grown for 2 weeks on 2,4‐D medium with or without (Mock) different concentrations of NPA, and subsequently grown for 1 week on medium without 2,4‐D or NPA. (c) The number of non‐fused (normal) somatic embryos (SEs) per 50 IZEs that were first grown for 2 weeks on 2,4‐D medium and subsequently grown for 1 week on medium without 2,4‐D and with different concentrations of NPA. The dots in (b) and (c) indicate the number of normal somatic embryos produced per 50 IZEs (*n* = 4 biological replicates), bars indicate the mean and error bars indicate the SEM. Different letters indicate statistically significant differences (*P* < 0.001) as determined by one‐way analysis of variance with Tukey's honest significant difference *post hoc* test. (d) The phenotypes of somatic embryos formed on cotyledons of a 2‐week‐old *p35S:AHL15* IZE on B5 medium supplemented with 20 μm NPA (lower) and without NPA (upper). (e) The expression pattern of *pWOX2:NLS‐YFP* in cotyledon tissues of *p35S:AHL15* IZEs after 7, 9 or 12 days of culture on medium without NPA (upper) or on medium supplemented with 20 μm NPA (lower). Images represent an overlay of the green (YFP) and red (chlorophyll) fluorescence. (a, d) Scale bars = 1 mm. (e) Scale bar = 0.5 mm.

To determine at which stage of SE auxin efflux is required, we analyzed the effect of the auxin efflux inhibitor *N*‐1‐naphthylphthalamic acid (NPA) on the induction of embryogenic cells and formation of pro‐embryos during the 14 days of culture on 2,4‐D containing medium (Figure S6). Our experiments showed that the number of somatic embryos is only slightly decreased following treatment with different concentrations of NPA during this first phase of 2,4‐D‐induced SE. Moreover, somatic embryos resembled those obtained on medium without NPA (Figure 4a,b). By contrast, when explants were transferred to medium with NPA after 14 days of 2,4‐D treatment (Figure S6), the number of normal, non‐fused somatic embryos was strongly reduced and aberrant embryo‐like structures developed (Figure 4a,c). Because we cannot exclude the possibility that the slight effect of NPA treatment during the first step of SE is caused by NPA accumulation persisting during the second step, we conclude that auxin efflux plays no or only a minor role in the induction of embryonic cells and formation of pro‐embryos, but, similar to that in zygotic embryogenesis, it has a major role later in embryo patterning and development.

The effect of NPA was also tested on *p35S:AHL15* IZE cultures, during which we also monitored the expression of the *pWOX2:NLS‐YFP* embryo identity reporter. NPA‐treated *p35S:AHL15* IZEs only developed a few aberrant embryos with fused cotyledons (Figure 4d), whereas many somatic embryos (approximately 10–20 per explant) were formed in the absence of NPA (Figure 4d). After 7 days of culture, *pWOX2:NLS‐YFP* expression was similar in the presence or the absence of NPA (Figure 4 e). After 9 and 12 days of culture, we observed the usual reduction in *pWOX2:NLS‐YFP* expression during further patterning and development of somatic embryos (Figure 4e), whereas high expression of *pWOX2:NLS‐YFP* persisted until day 12 in NPA‐treated AHL15‐induced somatic embryos (Figure 4e). The maintenance of *WOX2* expression might be caused by intracellular auxin accumulation as a result of a lack of efflux, similar to that observed for 2,4‐D‐cultured embryogenic calli. Taken together, these data indicate that in both 2,4‐D and AHL15‐induced SE, the maintenance but not the acquisition of embryonic identity requires high intracellular auxin. These steps proceed independently of auxin efflux, whereas the third step, further development and patterning, relies on auxin efflux to relocalize and focus intracellular auxin.

**Figure 5:**
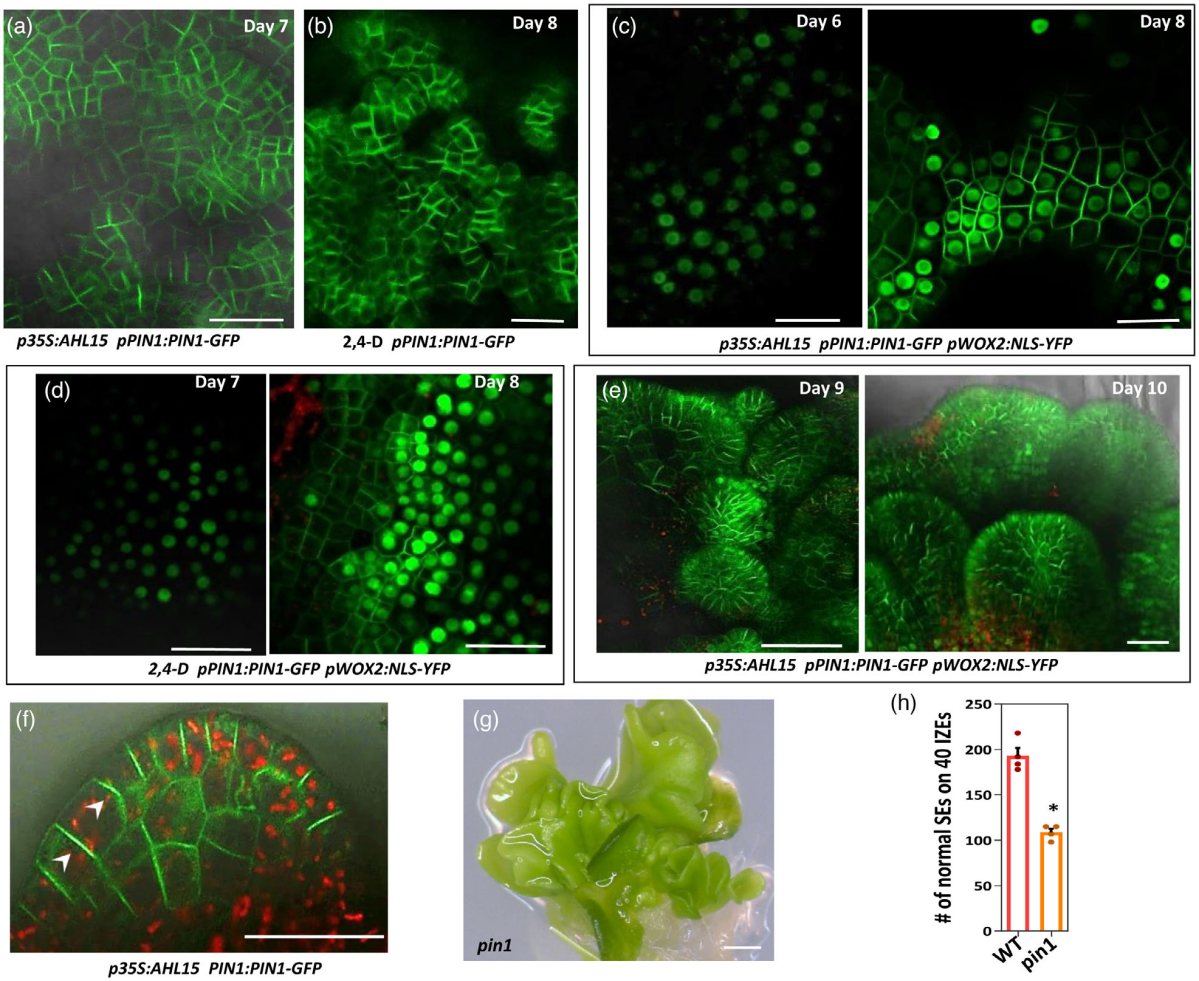
Expression and localization of *PIN1* during AHL15‐ and 2,4‐D‐induced SE. (a, b) PIN1‐GFP signals detected in cotyledons of *p35S:AHL15* IZEs after 7 days of culture (a) or in cotyledons of wild‐type IZEs after 8 days of culture on medium supplemented with 5 μm 2,4‐D (b). (c) Expression of *pPIN1:PIN1‐GFP* (plasma membrane) and *pWOX2:NLS‐YFP* (nucleus) in cotyledon tissues of *p35S:AHL15* IZEs after 6 days (left) or 8 days (right) of culture. (d) Expression of *pPIN1:PIN1‐GFP* and *pWOX2:NLS‐YFP* in cotyledon tissues of wild‐type IZEs after 7 days (left) or 8 days (right) of culture on medium supplemented with 5 μm 2,4‐D. In (c) (left) and (d) (left), note that the cotyledon cells show a clear nuclear WOX2‐YFP signal, whereas no PIN1‐GFP is visible. (e) Expression of *pPIN1:PIN1‐GFP* and *pWOX2:NLS‐YFP* in globular (left) and heart (right) stage embryos developing on cotyledons of *p35S:AHL15* IZEs cultured for respectively 9 or 10 days. (f) The expression of *PIN1:PIN1‐GFP* in globular‐heart stage somatic embryos on *p35S:AHL15* IZEs. Arrowheads indicate apical localization of PIN1‐GFP. Images in (d), (e) and (f) represent an overlay of the green (GFP) and red (chlorophyll) fluorescence. (g) The phenotype of somatic embryos formed on cotyledons of *pin1* mutant IZEs that were first grown for 2 weeks on 2,4‐D medium and subsequently cultured for 1 week on medium without 2,4‐D. (h) The number of non‐fused (normal) somatic embryos (SEs) per 40 WT or *pin1* mutant IZEs that were first grown for 2 weeks on 2,4‐D medium and subsequently grown for 1 week on medium without 2,4‐D. The dots indicate the number of normal somatic embryos (*n* = 4 biological replicates), bars indicate the mean and error bars indicate the SEM. Asterisk indicates significant differences from WT (*P < 0.01) as determined by a two‐sided Student's *t*‐test. (a–f) Scale bars = 100 μm. (g) Scale bar = 1 mm.

Polarly localized PIN proteins on the plasma membrane drive auxin efflux‐mediated patterning during zygotic embryogenesis (Friml et al., 2003). Therefore, we examined the expression of *pPIN1:PIN1‐GFP*, *pPIN2:PIN2‐VENUS, pPIN4:PIN4‐GFP* and *pPIN7:PIN7‐GFP* reporters during SE. Of these reporters, only PIN1‐GFP expression was observed in *35 S:AHL15* and 2,4‐D‐cultured IZE cotyledons, which was detected after 7–8 days of culture at the abaxial side of the cotyledons (Figure 5a,b). These results indicate that PIN1 is an important mediator of auxin efflux during *AHL15* and 2,4‐D‐induced SE.

To further monitor PIN1 activity during SE, co‐expression of *pWOX2:NLS‐YFP* and *pPIN1:PIN1‐GFP* were tracked in the *AHL15*‐ and 2,4‐D‐induced SE systems. Time‐lapse experiments showed that *pPIN1:PIN1‐GFP* expression is initiated 1–2 days after *WOX2* expression in the same cells in both systems (Figure 5c,d). During subsequent somatic embryo development (for the 2,4‐D system on medium without 2,4‐D), *pPIN1:PIN1‐GFP* expression was maintained in the embryo, but *pWOX2:NLS‐YFP* disappeared (Figure 5e). We did not observe clear polar localization of PIN1‐GFP in early embryonic cells during SE, whereas PIN1‐GFP localized apically in some cells in the globular stages (Figure 5f). Moreover, the number of normal (non‐fused) somatic embryos formed on 2,4‐D‐treated *pin1* mutant IZEs was significantly decreased compared to wild‐type Arabidopsis IZEs (Figure 5g,h), confirming the importance of PIN1 during somatic embryo patterning. These results, together with the auxin efflux inhibitor data, suggest that induction of embryonic cell identity does not require PIN1 function, but that PIN1 promotes the development of normal multicellular embryos from embryonic cells.

### Auxin influx is required for embryonic cell identity maintenance during SE

**Figure 6:**
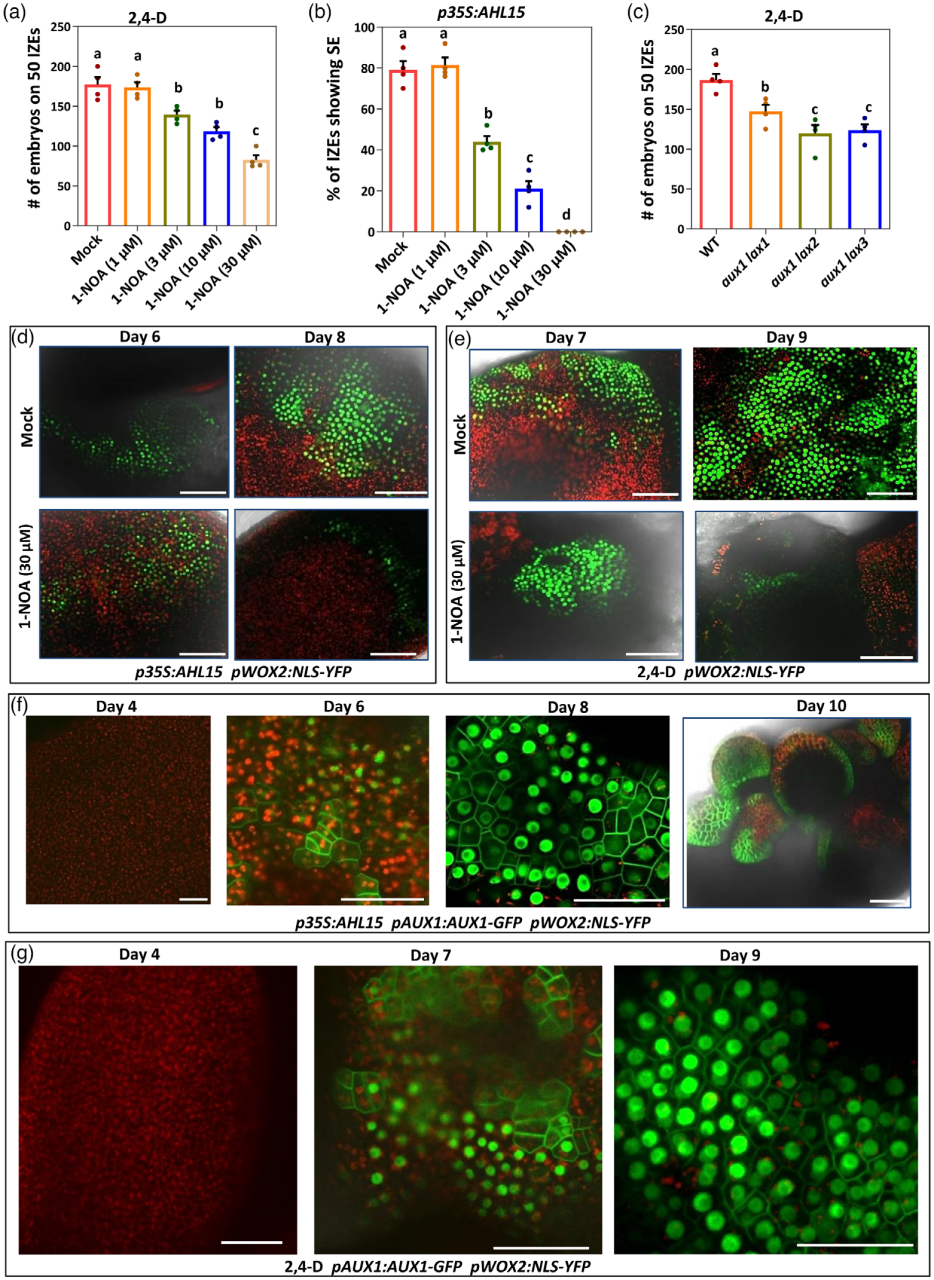
AUX1‐mediated auxin influx is required for embryonic cell identity maintenance during SE. (a) Number of somatic embryos per 50 wild‐type IZEs that develop after explants were first grown for 2 weeks on 2,4‐D medium with different concentrations of 1‐NOA, and subsequently grown for 1 week on medium without 2,4‐D (*n* = 4 biological replicates, with 50 IZEs per replicate) (b) Efficiency of embryo induction (% of 50 IZEs forming somatic embryos) on cotyledons of *p35S:AHL15* IZEs on medium with different concentrations of 1‐NOA. (c) The number of non‐fused somatic embryos (normal SEs) per 50 IZEs in WT, *aux1 lax1*, *aux1 lax2* or *aux1 lax4* mutant that were first grown for 2 weeks on 2,4‐D medium and subsequently grown for 1 week on medium without 2,4‐D. Dots in (a), (b) and (c) indicate the number or percentage and horizontal lines indicate the mean, error bars indicate the SEM. Different letters indicate statistically significant differences in (a) and (b) (*P* < 0.01) and in (c) (*P* < 0.05) as determined by one‐way analysis of variance with Tukey's honest significant difference *post hoc* test. (d) The expression pattern of *pWOX2:NLS‐YFP* in *p35S:AHL15* IZE cotyledons after 6 or 8 days of culture on medium without (upper) or with 30 μm 1‐NOA (lower). (e) The expression pattern of *pWOX2:NLS‐YFP* in cotyledons of wild‐ type IZEs after 6 or 9 days of culture on medium with 2,4‐D only (upper) or with 2,4‐D and 30 μm 1‐NOA (lower). (f) The expression patterns of *pAUX1:AUX1‐GFP* (plasma membrane) and *pWOX2:NLS‐YFP* (nucleus) in cotyledons of *p35S:AHL15* IZEs after 5, 6 or 8 days of culture or in globular to heart stage somatic embryos formed after 10 days of culture. (g) The expression patterns of *pAUX1:AUX1‐GFP* and *pWOX2:NLS‐YFP* in cotyledons of wild‐type IZEs after 4, 7 or 9 days of culture on medium with 2,4‐D. In (d–g), images represent an overlay of the green (YFP or GFP) and red (chlorophyll) fluorescence. Scale bars = 100 μm.

Auxin influx carriers facilitate the import of auxin into plant cells and play a critical role in the directional auxin flow and the resulting auxin maxima and minima formed during zygotic embryogenesis (Boot et al., 2016; Robert et al., 2015; Ugartechea‐Chirino et al., 2010). Adding the auxin influx inhibitor 1‐naphthoxyacetic acid (1‐NOA) (Parry et al., 2001) from the start of IZE culture in the 2,4‐D‐induced SE system strongly reduced the number of embryos formed on cotyledons (Figure 6a). As with yuc treatment, AHL15‐induced SE was significantly more sensitive to 1‐NOA treatment than 2,4‐D‐treated explants. Treatment with 30 μm 1‐NOA completely blocked the production of embryos on cotyledons of *p35S:AHL15* IZEs (Figure 6b), whereas this was not observed for 2,4‐D‐induced SE (Figure 6a). Incubation of *aux1 lax1*, *aux1 lax2* and *aux1 lax3* double mutant IZEs on 2,4‐D medium significantly reduced the number of somatic embryos compared to wild‐type Arabidopsis IZEs (Figure 6c). These findings corroborate our results with 1‐NOA treatment and show that the AUX1/LAX influx proteins act redundantly during SE and are required for embryonic cell identity maintenance.

To explore whether impaired auxin influx affects the initiation or maintenance of embryonic cell identity during SE, we tracked *pWOX2:NLS‐YFP* expression in time. Both in the presence or in the absence of 1‐NOA, *pWOX2:NLS‐YFP* expression was observed after 6–7 days of culture in *35:AHL15* (Figure 6d) and 2,4‐D‐treated IZE cotyledons (Figure 6e). However, 1–2 days later, the *pWOX2:NLS‐YFP* signals were greatly reduced in cotyledons of 1‐NOA‐treated IZEs compared to mock‐treated IZEs (Figure 6d,e). These results suggest that, as for auxin biosynthesis, auxin influx is not required for the first step of SE, initiation of embryo identity, but is required for the second step, embryonic identity maintenance. The negative effect of the yuc auxin biosynthesis inhibitor on *p35S:AHL15* IZEs could be complemented by providing exogenous IAA (Figure 2f). However, co‐treatment of *p35S:AHL15* IZEs with 20 μm yuc and 30 μm 1‐NOA disrupted the complementation by exogenously added IAA (Figure S7), confirming that embryonic cell identity maintenance relies on elevated intracellular IAA levels mediated by both auxin biosynthesis and auxin influx.

Analysis of *pAUX1:AUX1‐GFP* and *pLAX1:LAX1‐GFP* cultured IZEs showed that only AUX1‐GFP is expressed during 2,4‐D and AHL15‐induced SE. AUX1‐GFP signals coincide initially with the appearance of *pWOX2:NLS‐YFP* marked embryonic cells in cotyledons (day 6–8, Figure 6f,g), but, unlike *WOX2* expression, *AUX1* expression is maintained in globular embryos (day 10) (Figure 6f). These results suggest that the AUX1, LAX2 and LAX3 influx proteins act redundantly by mediating auxin uptake during the first two steps of SE, indicating that auxin influx and efflux balance the auxin levels in cells to maintain embryo identity and to promote embryo patterning.

## DISCUSSION

SE is a unique biological process in which differentiated somatic cells acquire embryo identity and develop into embryos. The mechanisms driving the acquisition of embryo cell fate in somatic cells comprise a fundamental question in plant biology. Although recent work has suggested that SE involves a complex signaling network and large‐scale transcriptional reprogramming (Wang et al., 2020), the molecular mechanisms underlying SE are not well understood. Given that an increase in endogenous auxin levels is often associated with efficient SE (Awada et al., 2019; Cheng et al., 2016; Ivanova et al., 1994; Jiménez & Bangerth, 2001a; Jiménez & Bangerth, 2001b; Márquez‐López et al., 2018; Michalczuk & Druart, 1999; Vondrakova et al., 2018), we investigated when and how endogenous auxin promotes SE using 2,4‐D and AHL15‐induced SE in Arabidopsis IZE explants.

### Auxin biosynthesis is required to maintain embryo identity during SE

*De novo* IAA biosynthesis in plant tissues has a large influence on plant growth and development and is essential for proper zygotic embryo patterning (Robert et al., 2013; Zhao, 2018). The increase in endogenous IAA levels during 2,4‐D‐induced SE is assumed to be required for somatic embryo formation and to be mediated by upregulation of the IPyA auxin biosynthesis route (Awada et al., 2019; Cheng et al., 2016; Ivanova et al., 1994; Jiménez & Bangerth, 2001a; Jiménez & Bangerth, 2001b; Márquez‐López et al., 2018; Michalczuk & Druart, 1999; Vondrakova et al., 2018). Recent data show that SE induced by the AINTEGUMENTA‐LIKE (AIL) transcription factor BABYBOOM (BBM) also requires YUC‐dependent auxin biosynthesis (Li et al., 2022).

Previous *YUC* reporter and *yuc* mutant analysis in 2,4‐D induced SE suggests that the *YUC1/2/4/6/10/11* genes are required during direct SE (SE without a callus phase) (Wójcikowska et al., 2013) and the *YUC1/2/4/6* genes are required during secondary SE (SE from somatic embryo‐derived callus) (Bai et al., 2013). In the BBM‐induced direct SE system, *YUC3* and *YUC8* were shown to act directly downstream of BBM and to be essential for efficient SE. Moreover application of the IAA biosynthesis inhibitor yuc (Nishimura et al., 2014) at different time points indicated that auxin biosynthesis is required for maintenance of embryo identity and growth (Li et al., 2022). In our indirect (embryogenic callus) 2,4‐D‐induced SE system, *YUC4/6/11* were upregulated together with *YUC7/8/9*. Application of yuc significantly reduced the number of 2,4‐D‐induced somatic embryos. *YUC6/7/8/9* gene expression was also found to be upregulated in somatic embryo‐forming cotyledons of cultured *p35S:AHL15* IZEs (also indirect SE). Moreover, application of the yuc or kyn IAA biosynthesis inhibitors severely impaired AHL15‐induced SE, whereas exogenous IAA application alleviated the repression of SE caused by yuc and kyn. Based on our data and given the well‐established correlation between the expression of *YUC* genes and IAA levels (Hentrich et al., 2013; Kim et al., 2011), we conclude that the elevated *YUC6*/*7*/*8*/*9* expression in cotyledons of cultured 2,4‐D‐treated or *35 S:AHL15* IZEs contributes to increased IAA levels, which are crucial for the development of somatic embryos on these tissues.

Recently, we showed that up‐regulation of *AHL* gene expression is required for 2,4‐D‐induced SE (Karami et al., 2021). Here, we show that the expression of the *YUC6/7/8/9* genes is not up‐regulated in response to 2,4‐D in the *ahl* loss‐of‐function mutant background. Therefore, *AHL* genes probably act downstream of 2,4‐D and upstream of *YUC*‐mediated auxin biosynthesis. Moreover, *AHL15* and other *AHL* genes were also shown to be direct targets of BBM and to act redundantly in BBM‐induced SE (Karami et al., 2021). Interestingly, *YUC8* is induced during both AHL15‐ and BBM‐induced SE, whereas *YUC3* is only induced in the BBM system (Li et al., 2022). These results suggest that AHL proteins act in concert with BBM with respect to activating the expression of specific *YUC* genes during 2,4‐D or BBM‐induced SE. In addition, the different data sets described above clearly show that distinct groups of *YUC* genes are activated and required to increase IAA levels in the different SE systems (direct, indirect or secondary SE). Expression of the auxin response *pDR5:GFP* reporter is clearly reduced in *ahl* loss‐of‐function mutant zygotic embryos (Figure S5b), suggesting that *AHL* genes, similar to *BBM* (Horstman et al., 2017), also have a role in triggering auxin biosynthesis in zygotic embryos. However, this requires further verification.

**Figure 7:**
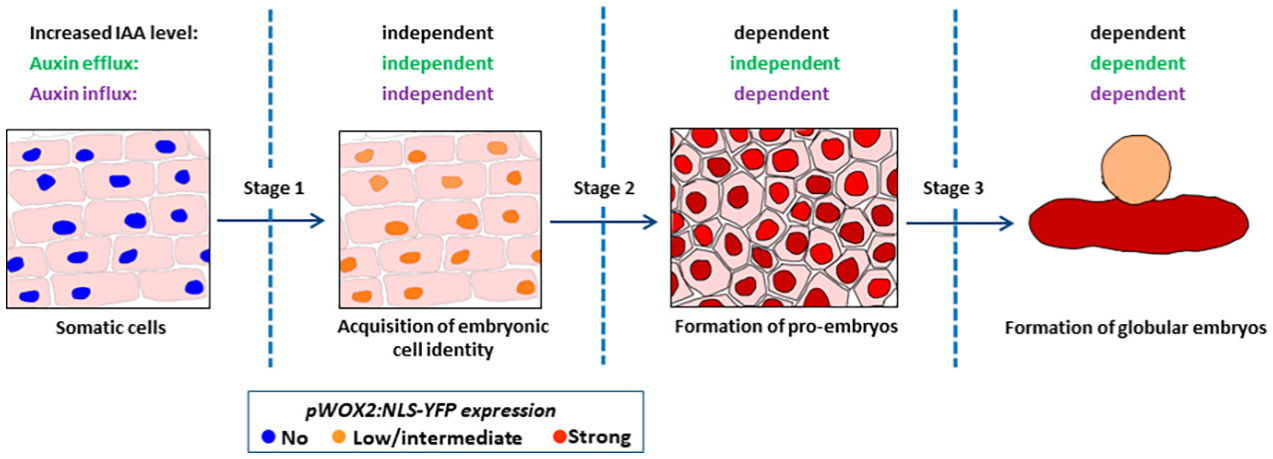
Model for the role of endogenous auxin during different stages of SE. The schematic diagram shows the three distinct developmental stages of somatic embryo induction on cotyledons of Arabidopsis IZEs, as distinguished by the *pWOX2:NLS‐YFP* embryonic cell identity reporter. The reporter is not expressed in IZEs at the start of culture. Low WOX2 expression marks the acquisition of embryo identity (stage 1), which occurs independent of endogenous auxin biosynthesis or transport. The maintenance of embryonic identity leading to the formation of pro‐embryos coincides with an increase in *pWOX2:NLS‐YFP* expression (stage 2) and requires auxin biosynthesis and auxin influx. The subsequent development of pro‐embryos into globular embryos and further embryo development is marked by the loss of *pWOX2:NLS‐YFP* expression (stage 3) and is dependent on auxin biosynthesis and on auxin transport by the efflux and influx machinery.

Our results show that *pWOX2:NLS‐YFP* expression marks three different stages of SE in IZE cotyledon tissues: (i) acquisition of embryo identity marked by low *pWOX2:NLS‐YFP* expression; (ii) maintenance of embryonic cell identity and development of pro‐embryos showing high *pWOX2:NLS‐YFP* expression; and (iii) development of pro‐embryos into globular embryos and further embryo patterning and development, coinciding with loss of *pWOX2:NLS‐YFP* expression (Figure 7; Figure S1c). By tracking the activity of this reporter in 2,4‐D treated and *p35S:AHL15* cotyledon cells, we show that induction of embryo identity in cotyledon cells does not require auxin biosynthesis because it occurs in the presence of the yuc or kyn inhibitor. However, under these conditions, embryo identity is not maintained following its acquisition, resulting in a rapid conversion to non‐embryonic cells. Therefore, we propose that elevated IAA levels are required for the maintenance of embryonic identity and progression of embryogenesis. This is in line with the critical role of YUC‐dependent auxin biosynthesis in zygotic embryo development and patterning (Cheng et al., 2006; Robert et al., 2013).

This conclusion immediately triggers two questions. (i) If endogenous auxin is not involved in the acquisition of embryo identity, how can SE be triggered by the auxin analog 2,4‐D? (ii) Why is 2,4‐D itself incapable of maintaining embryonic cell identity? The answer to the first question might be that acquisition of embryo identity requires reprogramming by extensive chromatin remodeling (Karami et al., 2021; Wang et al., 2020), something that can only be achieved by overexpression of chromatin/DNA binding proteins or by non‐physiological auxin levels. With respect to the second question, the low efficiency of polar cell‐to‐cell transport of 2,4‐D compared to IAA and the specific interaction of 2,4‐D with the auxin signaling machinery (Ma et al., 2018) might provide possible explanations.

### Auxin influx and efflux are required for maintaining embryonic cell identify and for embryo development

The directional transport of auxin, facilitated by both influx and efflux carriers, generates and maintains auxin gradients in tissues, and is known to play a crucial role in the establishment of the embryonic axis and the development of the zygotic embryo (Adamowski & Friml, 2015; Möller & Weijers, 2009). By contrast, the function of auxin efflux and influx in SE remains largely unknown. In the present study, we showed that the auxin influx and efflux machinery plays an important role in, respectively, the maintenance of embryonic cell identity and the proper development of somatic embryos.

By using the auxin efflux inhibitor NPA and by tracking expression of the *pWOX2:NLS‐YFP* reporter, we found that the first two steps in SE, the acquisition of embryonic identity and the formation of pro‐embryos do not depend on directional auxin efflux. Instead, NPA disrupts the transformation of pro‐embryos into differentiated embryos. By contrast to the normal downregulation of *pWOX2:NLS‐YFP* during somatic embryo development, *pWOX2:NLS‐YFP* activity was maintained in pro‐embryos on NPA‐containing medium. From these data, we conclude that auxin efflux promotes the development of embryonic cell clusters (pro‐embryos) to globular‐stage somatic embryos and later affects cell fate specification and differentiation during further embryo development.

Among the PIN1‐type proteins (PIN1/2/3/4/7) that facilitate auxin efflux in Arabidopsis (Adamowski & Friml, 2015), we only detected expression of PIN1 in embryonic cells and later during somatic embryo development (globular stage onward) (Figure 5). Previous studies have demonstrated that elevated auxin levels activate the expression of PIN1 proteins (Vieten et al., 2005). Therefore, the appearance of PINI in the embryonic cells may be associated with the auxin biosynthesis‐facilitated increase in auxin levels in these tissues.

During zygotic embryogenesis, the asymmetric localization of PIN1 on the plasma membrane plays an important role in auxin gradient formation, which is instrumental in cell type specification and pattern formation (Friml, 2010). PIN1 is expressed in early 16‐cell stage zygotic embryos, where it shows apolar localization. At the 32‐cell stage, however, it becomes polarly localized in the provascular tissue to generate an auxin maximum that specifies the hypophyseal cell group. Later, in globular‐stage embryos, PIN1 is asymmetrically localized at the plasma membrane of the upper apical region, producing auxin maxima that coincide with the formation of cotyledon primordia (Friml et al., 2003). We did not observe clear polar localization of PIN1 in early embryonic cells during SE, whereas its polar localization on the plasma membrane was detected in the globular and subsequent embryo stages. This suggests that, as in zygotic embryos, in the first embryogenic cell divisions, auxin is not initially polarly transported, but instead is evenly distributed over the embryonic cells. The question arises as to whether PIN1 is the only carrier that facilitates auxin efflux during SE? Other PIN proteins (e.g. PIN3) or other auxin transporters, such as the ATP‐binding cassette (ABC) auxin efflux transporters (Geisler et al., 2017), might also contribute to auxin distribution during SE.

Unlike NPA treatment, we found that embryo identity was rapidly lost in explants treated with the auxin influx inhibitor 1‐NOA. Auxin influx therefore appears to play a crucial role in the maintenance of embryonic cell identity. We suggest that loss of embryonic identity after 1‐NOA treatment is related to the reduction of auxin levels in embryonic cells. Using reporters for two of the four AUX/LAXs proteins that facilitate auxin influx (Swarup & Bhosale, 2019), we detected expression of *AUX1* during SE, but not of *LAX1*. Tests with different *aux1lax* double mutant combinations suggest that AUX1 acts redundantly with LAX2 and LAX3 during SE. Co‐expression of *AUX1* and *pWOX2:NLS‐YFP* in embryonic cells suggests that *AUX1* and *PIN1* co‐balance auxin influx and efflux in embryonic cells. We hypothesize that PIN1 or other auxin efflux carriers transport auxin to the extracellular space, whereas AUX1 prevents auxin leakage by transporting extracellular auxin back to the cytoplasm. Thus, the cooperation between auxin influx and efflux carriers in embryonic cells balances auxin levels to maintain embryonic cell identity.

## CONCLUSIONS

Taken together, our results show that endogenous auxin has different roles during the different stages of 2,4‐D‐ and AHL15‐induced SE. We show that the induction of embryonic identity proceeds independently of auxin biosynthesis and the auxin efflux and influx machinery. By contrast, auxin biosynthesis and auxin influx are essential for the maintenance of embryonic cell identity (Figure 7). Development of embryonic cells into pro‐embryos and their further development also requires an increase in auxin levels together with the auxin efflux and influx machinery (Figure 7). These findings can be used for the optimization of regeneration capacity via SE and for understanding the role of auxin signaling in the regulation of zygotic embryo patterning.

## EXPERIMENTAL PROCEDURES

### Plant material and growth conditions

All *A. thaliana* lines used in the present study were in the Columbia (Col‐o) background. The transgenic lines *p35S:AHL15, ahl15/+ pAHL15:AHL15‐GUS* (Karami et al., 2021), *pDR5:GFP* (Ottenschläger et al., 2003), *pWOX2:NLS‐YFP* (Breuninger et al., 2008), *pYUC1:NLS‐3xGFP*, *pYUC2:GFP‐GUS*, *pYUC3:NLS‐3xGFP*, *pYUC4:NLS‐3xGFP*, *pYUC5:GFP‐GUS*, *pYUC6:GFP‐GUS*, *pYUC7:GFP‐GUS*, *pYUC8:GFP‐GUS*, *pYUC9:GFP‐GUS*, *pYUC10:GFP‐GUS*, *pYUC11:GFP‐GUS* (Robert et al., 2013), *pPIN1:PIN1‐YFP* (Benkova et al., 2003) *pAUX1:AUX1‐YFP* (Swarup et al., 2005) and *pLAX1:LAX1‐YFP* (Péret et al., 2012 and the *pin1* mutant Huang et al., 2010 and the *aux1 lax* double mutants (Bainbridge et al., 2008; Péret et al., 2012)) have been described previously. Seeds were sterilized in 10% (v/v) sodium hypochlorite for 12 min and then washed four times in sterile water. Sterilized seeds were plated on half MS medium (Murashige & Skoog, 1962) containing 1% (w/v) sucrose and 0.7% agar. Seedlings, plants and explants were grown under a 16‐h photoperiod at 21°C and 70% relative humidity.

### Somatic embryogenesis

For the isolation of IZEs at the bent cotyledon stage of development, siliques were harvested 10–12 days after pollination, sterilized in 10% (v/v) sodium hypochlorite for 7 min and then washed four times in sterile water. IZEs were dissected from the siliques inside a laminar flow cabinet (Gaj, 2001). In the AHL15‐induced SE system, p35S*:AHL15* IZEs were cultured on solid B5 (Gamborg et al., 1968) supplemented with 2% (w/v) sucrose and 0.7% agar (Sigma, St Louis, MO, USA) for 2 weeks under a 16‐h photoperiod at 21°C and 70% relative humidity. Two weeks after culture, the efficiency of SE induction was scored under a stereomicroscope as the percentage of 50 *p35S:AHL15* IZE explants per plate producing somatic embryos. Four plates were scored for each experiment. In the 2,4‐D‐induced SE system, wild‐type IZEs were cultured on solid B5 medium supplemented with 4.5 μm 2,4‐D, 2% (w/v) sucrose and 0.7% agar (Sigma) for 2 weeks. Subsequently, the embryonic structures were allowed to develop further by transferring the explants to half MS medium with 1% (w/v) sucrose and 0.7% agar (Sigma) without 2,4‐D. One week after subculture, the capacity to induce SE was scored under a stereomicroscope as the number of somatic embryos produced from 50 IZEs per plate. Four plates were scored for each experiment.

For NPA or 1‐NOA treatment, IZEs were cultured immediately after dissection on solid B5 medium supplemented with the indicated concentration of NPA or 1‐NOA. However, when IZEs were cultured immediately after dissection on B5 medium with yuc, all IZEs died. We therefore precultured the IZEs for 1 day on medium without yuc after which they were transferred to medium with yuc. All compounds were dissolved in DMSO at a concentration so that the appropriate concentration in the medium was obtained by a 1:1000 dilution. Mock‐treatments received the same volume of DMSO.

### GUS staining

Histochemical staining of transgenic lines expressing the β‐glucuronidase (GUS) reporter for GUS activity was performed as described previously (Anandalakshmi et al., 1998) for 4 h at 37°C, followed by rehydration in a graded ethanol series (75, 50 and 25%) for 10 min each.

### qPCR analysis

RNA was isolated from IZEs cultured on B5 medium with or without 2,4‐D using a RNEasy© Kit (Qiagen, Valencia, CA, USA). First‐strand cDNA was synthesized using the RevertAid RT Reverse Transcription Kit (Thermo Fisher Scientific, Walthem, MA, USA). Quantitative PCR was performed on three biological replicates along with three technical replicates using the SYBR‐green dye premixed master‐mix (Thermo Fischer Scientific) in a C1000 Touch© (Bio‐Rad, Hercules, CA, USA) thermal cycler. CT values were obtained using cfx manager 3.1 (Bio‐Rad). The relative expression level of genes was calculated according to the 2^−ΔΔCt^ method (Livak & Schmittgen, 2001). Expression was normalized using the *b‐TUBULIN‐6* gene as reference. The primers used are listed in Table S1.

### Microscopy

GUS‐stained tissues and cultured IZE explants were observed and photographed using a MZ12 microscope (Leica, Wetzlar, Germany) equipped with a DC500 camera (Leica).

Confocal laser scanning microscopy was performed with a LSM510 (Zeiss, Oberkochen, Germany) exciter equipped with argon and helium/neon lasers. GFP and YFP were detected using a 488 nm band pass filter for excitation and a 500–530 nm band pass filter for emission. Simultaneously, background fluorescence (e.g. of chlorophyll) was detected with a 650 nm long pass emission filter. Images were captured with zen2009 (Zeiss). The nuclear or tissue‐specific YFP or GFP intensity was quantified as mean gray values by analyzing images of independent samples capturing the same region of interest using imagej (NIH, Bethesda, USA), as previously described by Béziat et al. (2017). Unmodified images were cropped (if needed) and used for assembly into figures in powerpoint (Microsoft Corp., Redmond, WA, USA). Assembled figures were saved as pdf files and converted to tif files in photoshop (Adobe, San Jose, CA, USA).

### Data processing and statistical analysis

Phenotype scoring and quantification data were collected in excel (Microsoft Corp.) and from there imported into prism (GraphPad Software Inc., San Diego, CA, USA) for statistical analysis and to generate graphs. For one‐on‐one comparisons, the Student's *t*‐test was used. For intercomparison of more than two data points, one‐way analysis of variance with Tukey's honestly significant difference *post hoc* test was used. *P* < 0.05 was considered statistically significant.

## CONFLICT OF INTEREST

The authors declare that they have no competing interests.

## AUTHOR CONTRIBUTIONS

OK, KB and RO conceived the project. RO and KB obtained funding for the project. RO supervised the project. CP set up the 2,4‐D‐induced SE system and performed the initial experiments. OK performed most of the subsequent experiments. AR analyzed the expression of *YUCCA* genes in the AHL15‐induced SE system. ARN analyzed the effect of auxin biosynthesis inhibitors on both SE systems. OK, KB and RO wrote the manuscript and all authors read, commented on and agreed with the version submitted for publication.

## Supporting information


**Figure S1.** The *pWOX2:NLS‐YFP* reporter marks the early stages of ZE and SE.
**Figure S2.** *pWOX2‐NLS‐YFP* expression during AHL15‐induced SE.
**Figure S3.** *YUC* expression in Arabidopsis IZEs during culture on 2,4‐D‐medium.
**Figure S4.** *YUC* expression in Arabidopsis IZEs during AHL15‐induced SE.
**Figure S5.** *ahl* loss‐of‐function leads to a reduced auxin response and defects in zygotic embryos.
**Figure S6.** Schematic representation of the experimental setup to analyze the effect NPA on 2,4‐D‐induced SE.
**Figure S7.** Exogenous auxin requires auxin influx to rescue inhibition of auxin biosynthesis during AHL15‐induced SE.
**Table S1.** Primers used for qRT‐PCR.

## Data Availability

All relevant data can be found within the published article and its supporting material. Raw data and materials are available from RO upon reasonable request.
