# Supplementary material for: Endogenous auxin maintains embryonic cell identity and promotes somatic embryo development in Arabidopsis
Source: Plant J. 2022 Nov 28;113(1):7–22. doi: 10.1111/tpj.16024 (PMC10098609; doi:10.1111/tpj.16024)
Supplement: Supplementary file 1 — Figure S1. The pWOX2:NLS‐YFP reporter marks the early stages of ZE and SE. Figure S2. pWOX2‐NLS‐YFP expression during AHL15‐induced SE. Figure S3. YUC expression in Arabidopsis IZEs during culture on 2,4‐D‐medium. Figure S4. YUC expression in Arabidopsis IZEs during AHL15‐induced SE. Figure S5. ahl loss‐of‐function leads to a reduced auxin response and defects in zygotic embryos. Figure S6. Schematic representation of the experimental setup to analyze the effect NPA on 2,4‐D‐induced SE. Figure S7. Exogenous auxin requires auxin influx to rescue inhibition of auxin biosynthesis during AHL15‐induced SE. Table S1. Primers used for qRT‐PCR. [file TPJ-113-7-s001.docx]

**Supplemental Material**

**Endogenous auxin maintains embryonic cell identity and promotes somatic embryo development in Arabidopsis**

Omid Karami^1^, Cheryl Philipsen^1,3^, Arezoo Rahimi^1^, Annisa Ratna Nurillah^1,4^, Kim Boutilier^2^, and Remko Offringa^1,*^

**^1^**Plant Developmental Genetics, Institute of Biology Leiden, Leiden University, Sylviusweg 72, 2333 BE Leiden, Netherlands

^2^Bioscience, Wageningen University and Research, Droevendaalsesteeg 1, 6708 PB Wageningen, Netherlands

^3^ Current affiliation: Plus Projects, Zwaardstraat 16, 2584 TX The Hague, Netherlands

^4^ Current affiliation: BearingPoint Caribbean, Kaya Flamboyan 7, Willemstad, Curaçao, AN

*Correspondence should be addressed to R.O. (r.offringa@biology.leidenuniv.nl)

**
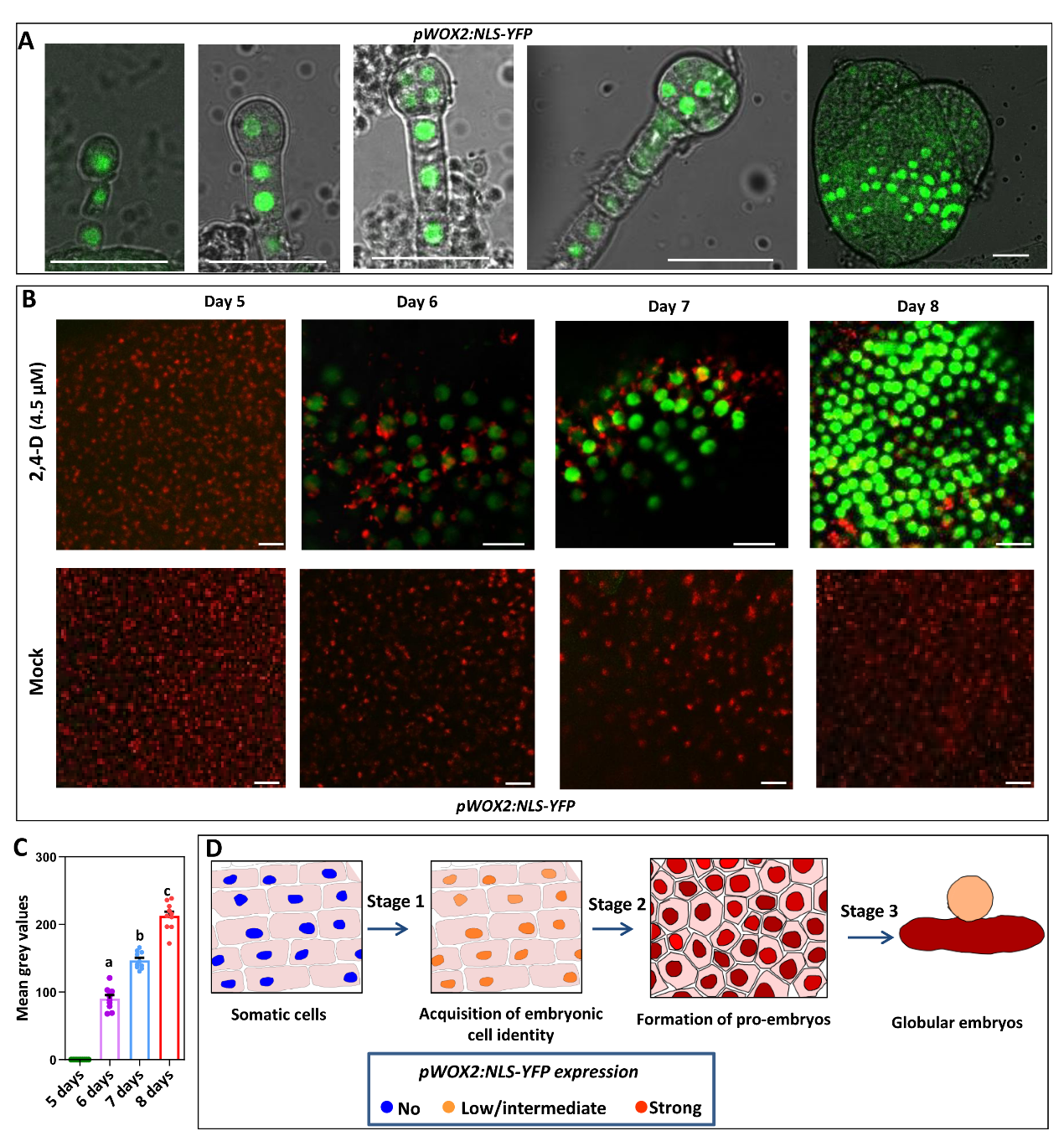
**

**Supplemental Figure 1.** **The *pWOX2:NLS-YFP* reporter marks early stages of ZE and SE.** (**A**) The *pWOX2:NLS-YFP* reporter is expressed in the embryo proper and suspensor during early stages of ZE. Size bar indicates 50 μm (**B**) The expression of *pWOX2:NLS-YFP* in cotyledon tissues of IZEs after four, six, seven and eight days on B5 medium supplemented with 2,4-D (up panel) or without 2,4-D (down panel). Size bars indicate 30 µm. Images represent an overlay of the green (YFP) and red (chlorophyll) fluorescence. (**C**) Quantification of YFP intensity (mean grey values) in *pWOX2:NLS-YFP* cotyledon tissues of IZEs after five, six, seven and eight days on B5 medium supplemented with 2,4-D. Dots indicate the values of ten biological replicates, with about 100 nuclei analyzed per replicate, bar indicates the mean, and error bars the s.e.m.. Different letters indicate statistically significant differences (P < 0.01) as determined by a one-way ANOVA with Tukey’s post hoc test. (**D**) A schematic representation of the three developmental stages of somatic embryo induction from Arabidopsis IZE explants that are distinguished by *pWOX2:NLS-YFP* expression: 1) acquisition of embryo identity in somatic cells around day 6, 2) rapid cell proliferation coinciding with the conversion of embryonic cells to pro-embryos around day 8 and 3) the development of pro-embryos into globular embryos around day 10 of culture in the AHL15-induced SE system, or following transfer of the explants to hormone free medium in the 2,4-D-induced SE system..


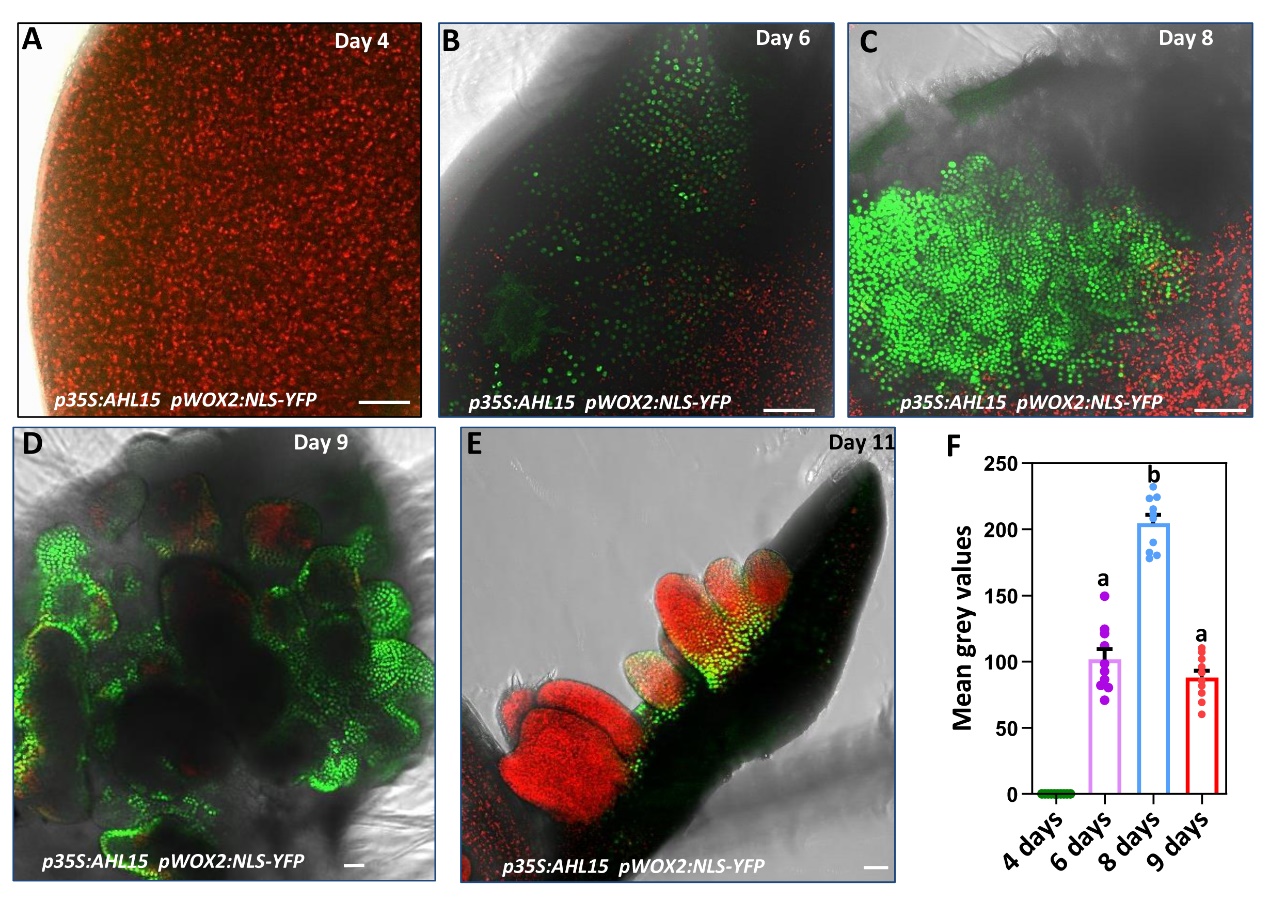
**Supplemental Figure 2. *pWOX2-NLS-YFP* expression during AHL15-induced SE.** (**A**-**C**) *pWOX2:NLS-YFP* expression in cotyledons of *p35S:AHL15* IZEs after four (**A**), six (**B**) and eight (**C**) days of culture. Note that the weak expression is first observed after six days of culture (**D** and **E**). *pWOX2:NLS-YFP* expression in globular (**D**) and torpedo (**E**) stage somatic embryos formed on cotyledons of *35S:AHL15* IZEs after nine (**D**) and eleven (**E**) days of culture. Size bars indicate 100 µm in **A**-**E.** Images represent an overlay of the green (YFP) and red (chlorophyll) fluorescence. (**F**) Quantification of YFP intensity (mean grey values) in *p35S:AHL15 pWOX2:NLS-YFP* cotyledon tissues of IZEs after four, six, eight and nine days on B5 medium supplemented with 2,4-D. Dots indicate the values of ten biological replicates, with about 100 nuclei analyzed per replicate, bar indicates the mean, and error bars the s.e.m.. Different letters indicate statistically significant differences (p < 0.01) as determined by a one-way ANOVA with Tukey’s post hoc test.

**
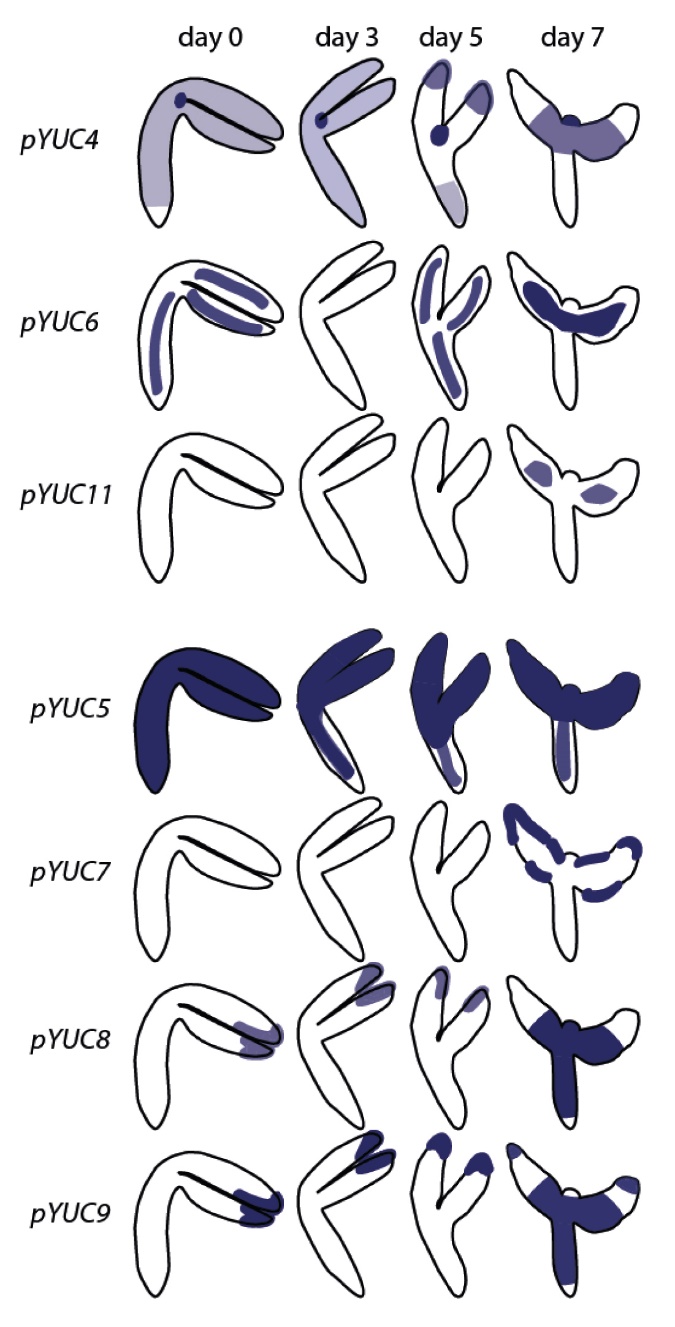
**

**Supplemental Figure 3. *YUC* expression in Arabidopsis IZEs during culture on 2,4-D-medium.** Schematic representation of *YUC* gene expression as monitored in wild-type Arabidopsis IZEs at zero, three, five or seven days of culture on medium with 4.5 µM 2,4-D using *YUC* promoter GFP-GUS lines and histochemical staining for GUS activity. The *YUC2*- and *YUC10* promoter-reporters did not show expression and are therefore not shown.


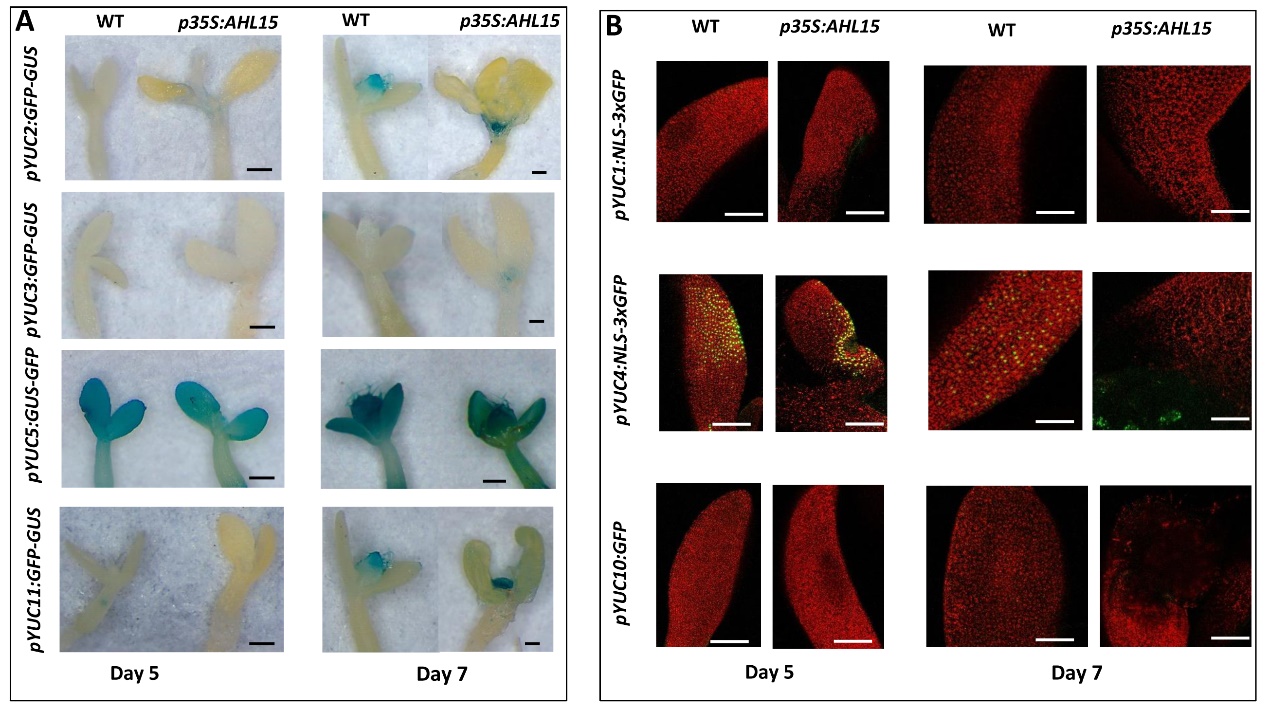


**Supplemental Figure 4.** ***YUC* expression in Arabidopsis IZEs during AHL15-induced SE** (**A**) Expression pattern of *pYUC2:GFP-GUS*, *pYUC3:GFP-GUS*, *pYUC5:**GFP-GUS* or *pYUC11:GFP-GUS* reporters in wild-type and *35S:AHL15* IZEs cultured for five (right) or seven (left) days on medium without 2,4-D. (**B**) Expression pattern of *pYUC1:NLS-GFP*, *pYUC4:NLS-GFP* or *pYUC10:GFP* reporters in wild-type and *p35S:AHL15* IZEs cultured for five (right) or seven (left) days on medium without 2,4-D. Scale bars in **A** and **B** indicate 1 mm.


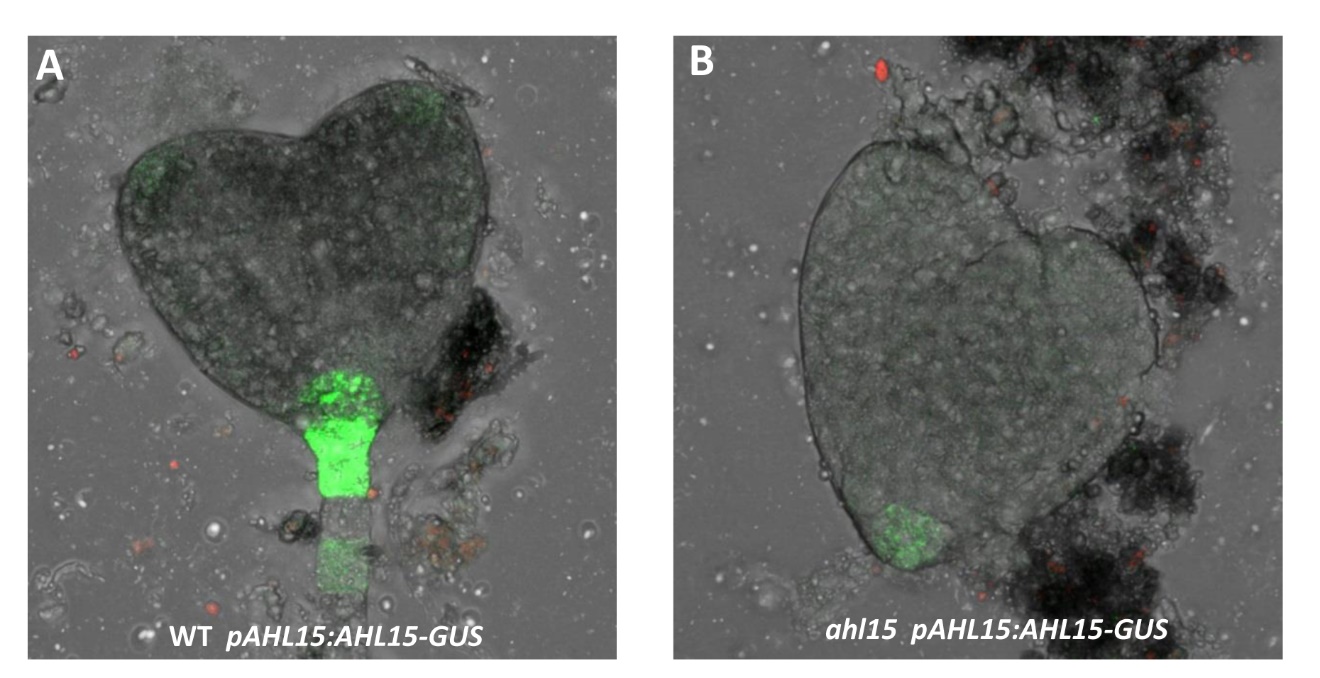


**Supplemental Figure 5. *ahl* loss-of-function leads to a reduced auxin response and defects in zygotic embryos.** (**A** and **B)** The expression pattern of *pDR5:GFP* reporter in wild-type (**A**) and *ahl15 pAHL15:AHL15-GUS* (**B**) zygotic embryos.


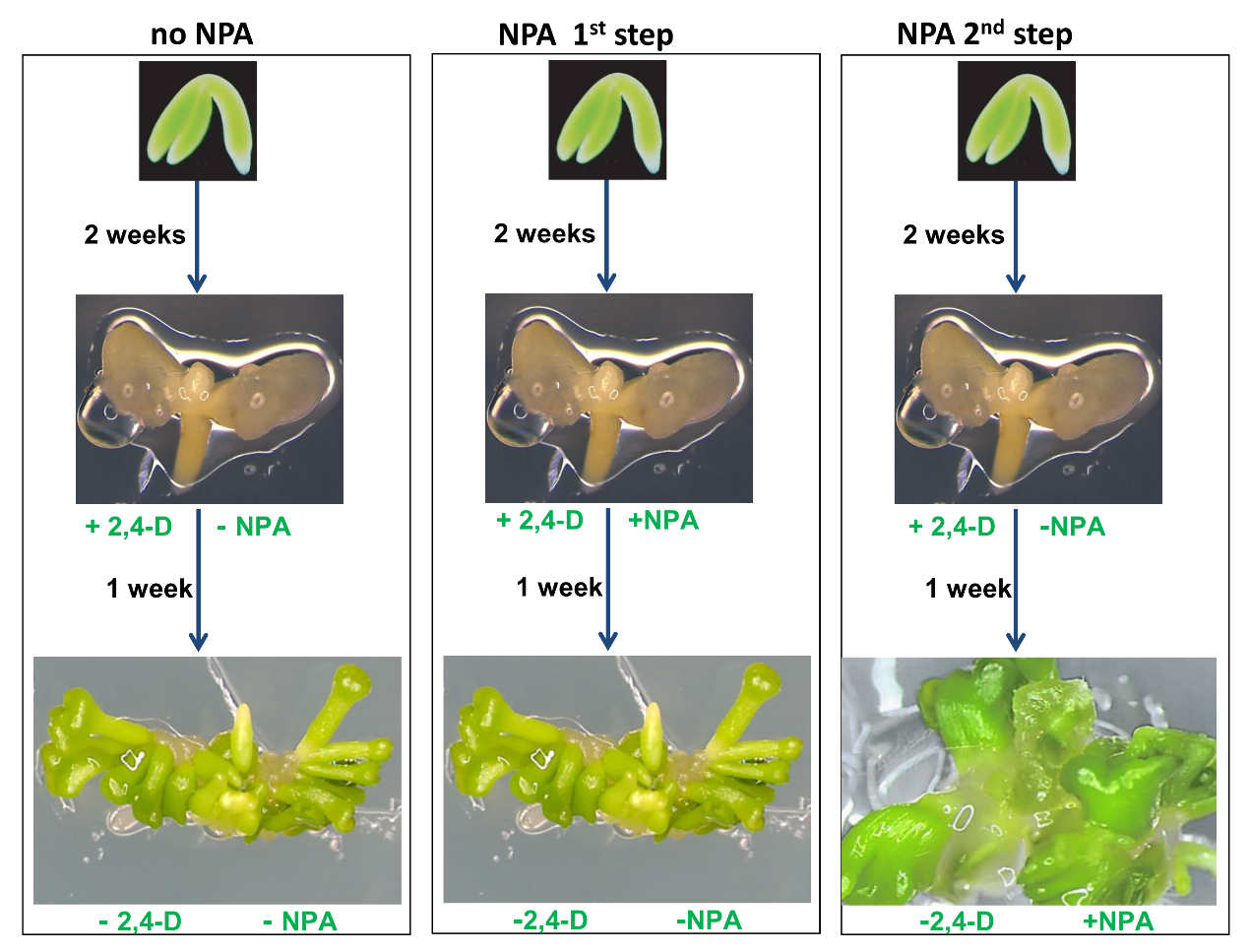


**Supplemental Figure 6.** Schematic representation of the experimental setup to analyze the effect NPA on 2,4-D-induced SE.


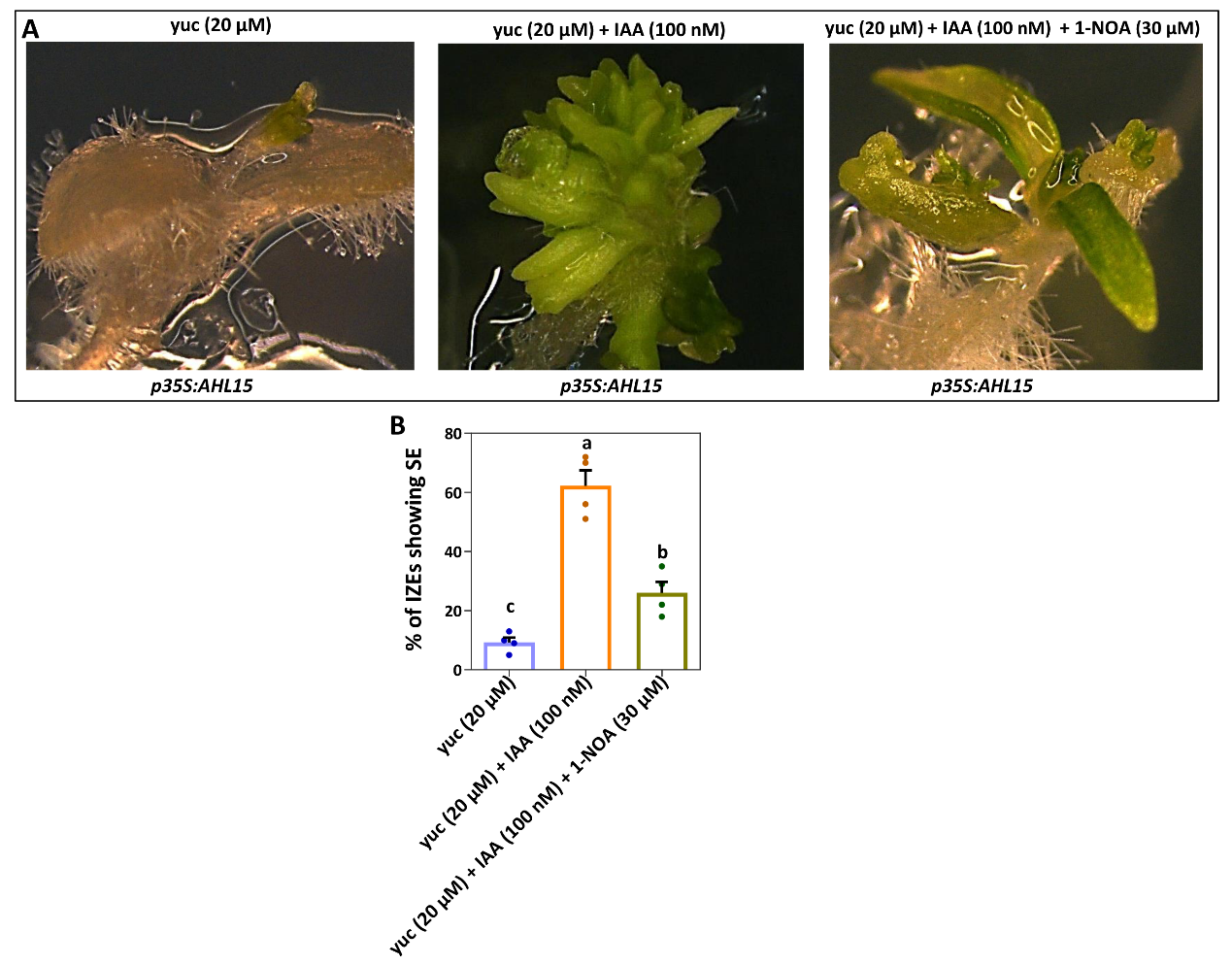


**Supplemental Figure 7**. **Exogenous auxin requires auxin influx to rescue inhibition of auxin biosynthesis during AHL15-induced SE.** (**A**) The phenotypes of *p35S:AHL15* IZEs cultured for two weeks on B5 medium supplemented with 20 µM yuc (left), 20 µM yuc with 100 nM IAA (middle) and 20 µM yuc with 100 nM IAA and 30 µM 1-NOA (right). (**B**) Efficiency of embryo induction (% of 35 IZEs forming somatic embryos) on *p35S:AHL15* cotyledons on B5 medium supplemented with 20 µM yuc, 20 µM yuc with 100 nM IAA and 20 µM yuc with 100 nM IAA and 30 µM 1-NOA. Dots indicate the percentage, horizontal lines indicate the mean and error bars indicate s.e.m. and different letters indicate statistically significant differences (P < 0.001) as determined by one-way analysis of variance with Tukey’s honest significant difference post hoc test.

**Table S1.** Primers used for qRT-PCR

| Name* | Sequence (5’ to 3’) | | Purpose |
| --- | --- | --- | --- |
| q YUC6-F | AAACTCCGGTTCTCGACGTTGG | qRT-PCR *YUC6* | |
| q YUC6-R | CCCGAACACACCTTAATGTCTCC |  |  |
| q YUC7-F | GGATGGTCGAGTTCTGCAGATTG | qRT-PCR  *YUC7* | |
| q YUC7-R | TCCTTAAGCCATGAAGGGACATTG |  | |
| q YUC8-F | TGCGGTTGGGTTTACGAGGAAAG | qRT-PCR  *YUC8* | |
| q YUC8-R | GCGATCTTAACCGCGTCCATTG |  | |
| q YUC9-F | ATTATACGCGGCCGGATTCACG | qRT-PCR *YUC9* | |
| q YUC9-R | AGCGATGTTAACGGCGTCTACTG |  | |
| qβ-TUBULIN-6-F | TGGGAACTCTGCTCATATCT | qRT-PCR *TUBULIN-6* | |
| qβ-TUBULIN-6-R | GAAAGGAATGAG GTTCACTG |  | |
|  |  |  | |

*, F: forward; R: reverse.
